# Flavonoids against depression: a comprehensive review of literature

**DOI:** 10.3389/fphar.2024.1411168

**Published:** 2024-10-16

**Authors:** Alaleh Alizadeh, Yeganeh Pourfallah-Taft, Maryam Khoshnazar, Aysan Safdari, Saba Vafadar Komari, Mehrnaz Zanganeh, Nafiseh Sami, Maryam Valizadeh, Arezoo Faridzadeh, Dorsa Alijanzadeh, Seyed Amirhossein Mazhari, Reza Khademi, Ali Kheirandish, Mahdyieh Naziri

**Affiliations:** ^1^ Student Research Committee, Faculty of Medicine, Mashhad Branch, Islamic Azad University, Mashhad, Iran; ^2^ Student’s Research Committee, School of Medicine, Shahid Beheshti University of Medical Sciences, Tehran, Iran; ^3^ Student Research Committee, School of Medicine, Mazandaran University of Medical Sciences, Sari, Iran; ^4^ Student Research Committee, Faculty of Nursing and Midwifery, Tabriz Branch, Islamic Azad University, Tabriz, Iran; ^5^ Student Research Committee, Tehran Medical Sciences Branch, Islamic Azad University, Tehran, Iran; ^6^ Dental Research Center, Mashhad University of Medical Sciences, Mashhad, Iran; ^7^ Department of Immunology and Allergy, School of Medicine, Mashhad University of Medical Sciences, Mashhad, Iran; ^8^ Immunology Research Center, Mashhad University of Medical Sciences, Mashhad, Iran; ^9^ Student Research Committee, Azerbaijan Medical University, Baku, Azerbaijan; ^10^ Student Research Committee, Faculty of Medicine, Mashhad University of Medical Sciences, Mashhad, Iran; ^11^ Student Research Committee, Faculty of Pharmacy, Mazandaran University of Medical Sciences, Sari, Iran; ^12^ Students Research Committee, School of Medicine, Iran University of Medical Sciences, Tehran, Iran

**Keywords:** tehran hemat highway next to milad Tower14535, Iran antidepressant, flavonoids, natural products, herbal medicine, complementary medicineIntroduction, depression

## Abstract

**Background:**

Depression is a state of low mood and aversion to activity, which affects a person’s thoughts, behavior, motivation, feelings, and sense of wellbeing. Pharmacologic therapies are still the best effective treatment of depression. Still, most antidepressant drugs have low efficacy and delayed onset of therapeutic action, have different side effects, and even exacerbate depression. Such conditions make it possible to look for alternatives. Consequently, we decided to summarize the impact of flavonoids on depression in this review.

**Methods:**

We searched scientific databases such as SCOPUS, PubMed, and Google Scholar to find relevant studies until July 2022.

**Results:**

A wide variety of natural components have been shown to alleviate depression, one of which is flavonoids. Due to the growing tendency to use natural antidepressant drugs, scientific studies are increasingly being conducted on flavonoids. This study aims to review the latest scientific researches that indicate the antidepressant potential of flavonoids. Various mechanisms include neurotransmitter system modulation and dopaminergic, noradrenergic, and serotonergic pathways regulation in the central nervous system. Different compounds of flavonoids have antidepressant properties *in vivo* or *in vitro* experiments or clinical trials and can be used as alternative and complementary treatments for depression. In general, it was observed that there were no severe side effects.

**Conclusion:**

Our study proves the antidepressant potential of flavonoids, and considering the limited side effects, they can be used as complementary medicine for depressed patients.

## 1 Introduction

Depression is a psychiatric disorder characterized by persistent sadness and a lack of interest or pleasure in previously rewarding or enjoyable activities. It can also disturb sleep and appetite. Tiredness and poor concentration are common. Depression is a leading cause of disability worldwide and contributes significantly to the global disease burden. The World Health Organization (WHO) estimated that the total number of people with depression was more than 300 million in 2015 (OrganizationWorld Health, 2017). Moreover, the WHO predicts that depression will be the primary cause of disability by 2030, putting a substantial social and financial burden on communities; in the first year of the COVID-19 pandemic, the global outbreak of anxiety and depression raised by a massive 25%, according to a scientific brief released by the WHO today. Despite exercise therapy, psychotherapy, and electroconvulsive therapy, pharmacologic therapies are still the best effective treatment for depression (Kok and Charles, 2017). A broad spectrum of antidepressant drugs, such as selective serotonin reuptake inhibitors (SSRI), tricyclic antidepressants, selective dopamine reuptake inhibitors, and selective norepinephrine reuptake inhibitors (SNRI), is introduced to change serotonergic, dopaminergic, glutamatergic, or noradrenergic activity levels in the central nervous system (CNS) (Harmer, Duman, and Cowen, 2017). Clinical response to these drugs depends on the drug’s pharmacodynamics and pharmacokinetic properties and the underlying biology of patients (Preskorn and Drevets, 2009).

## 2 Adverse reactions of antidepressant treatment

Most antidepressant drugs have low efficacy (about 40%–60%), different side effects, and can even exacerbate depression (Perez-Caballero et al., 2019; Santarsieri and Schwartz, 2015). Specific complications of tricyclic antidepressants can include sexual dysfunction, inability to drive, high blood pressure, blurred vision, dry mouth, thirst, constipation, dizziness, and distress (Dwyer, Whitten, and Hawrelak, 2011). Monoamine oxidase inhibitors (MAOIs) cause an increase in developing orthostatic hypotension and behavioral stimulation (Calvi et al., 2021). Some other adverse effects, such as hyponatremia, bleeding, and sexual dysfunction, are more prominent with either SNRIs or SSRIs (Wang et al., 2018). Studies show that sexual dysfunction develops during or after antidepressant therapy and persists after the depression is resolved, and the drug is discontinued (Rothmore, 2020). Although clinicians widely prescribe drug therapy for depression, fewer than half of the individuals treated with antidepressant medication respond to these drugs (Arteaga-Henríquez et al., 2019; Furukawa et al., 2016; Cipriani et al., 2009). Therefore, studies show that patients’ recovery is partial or incomplete, and some resist pharmacotherapy (Sforzini et al., 2022). These conditions develop an opportunity for alternative therapy for depression through medicinal plants (Wang et al., 2019). Various natural chemical agents have been shown to alleviate depression (Wang et al., 2018), one of which is flavonoids.

### 2.1 Flavonoids and depression: an overview

Flavonoids, a group of polyphenolic structures, are found abundantly in plants, especially fruits, vegetables, and medical herbs (Moon, Wang, and Morris, 2006). They are well known for their antioxidant activity as a dietary component (Kumar and Pandey, 2013). Furthermore, flavonoids are effective in many diseases with different mechanisms; for example, anticholinesterase activity in Alzheimer’s disease (AD) and steroidogenesis modulators in hormone-dependent cancer (Panche, Diwan, and Chandra, 2016a).

The main focus of some existing reviews (Ali S. et al., 2021; Jia et al., 2022; Khan et al., 2018; Ko et al., 2020; Pannu A. et al., 2021) are animal or human studies, however, the possible mechanisms have not been sufficiently investigated. In an article by Lucian Hritcu et al. (Hritcu et al., 2017a), animal.

Studies in the last 6 years are included. In a study published by Pannu et al. (Pannu Arzoo et al., 2021), only preclinical studies were included, and the effect of flavonoids on human studies was not examined. Other studies, such as clinical trials, found significant results of flavonoids on depression symptoms (Atteritano et al., 2014; Zarghami et al., 2018; Ali Sawan et al., 2021). In an article published by Yong-Hyun Ko et al. (Ko et al., 2020), other types of flavonoids were examined, while one of the most prominent features of the present article, as far as we know, is that none of the existing pieces in this field has examined all the groups and subgroups of flavonoids and their mechanisms as much as this study. The summary of the antidepressant properties of flavonoids is shown in Supplementary Table S1. Flavonoids can be broadly classified according to the carbon located in the B ring’s connection to the C ring. By the structural characteristics of the C ring, the flavonoids linked in position 3 of the C ring are known as isoflavones, those linked in position 4 are neoflavonoids, and those related to position two are divided into various subgroups (chalcones, anthocyanins, flavanols, flavanonols, flavanones, flavonols, and flavones) (Ramesh et al., 2021; Khan et al., 2021).

## 3 Chalcones

**FIGURE 1:**
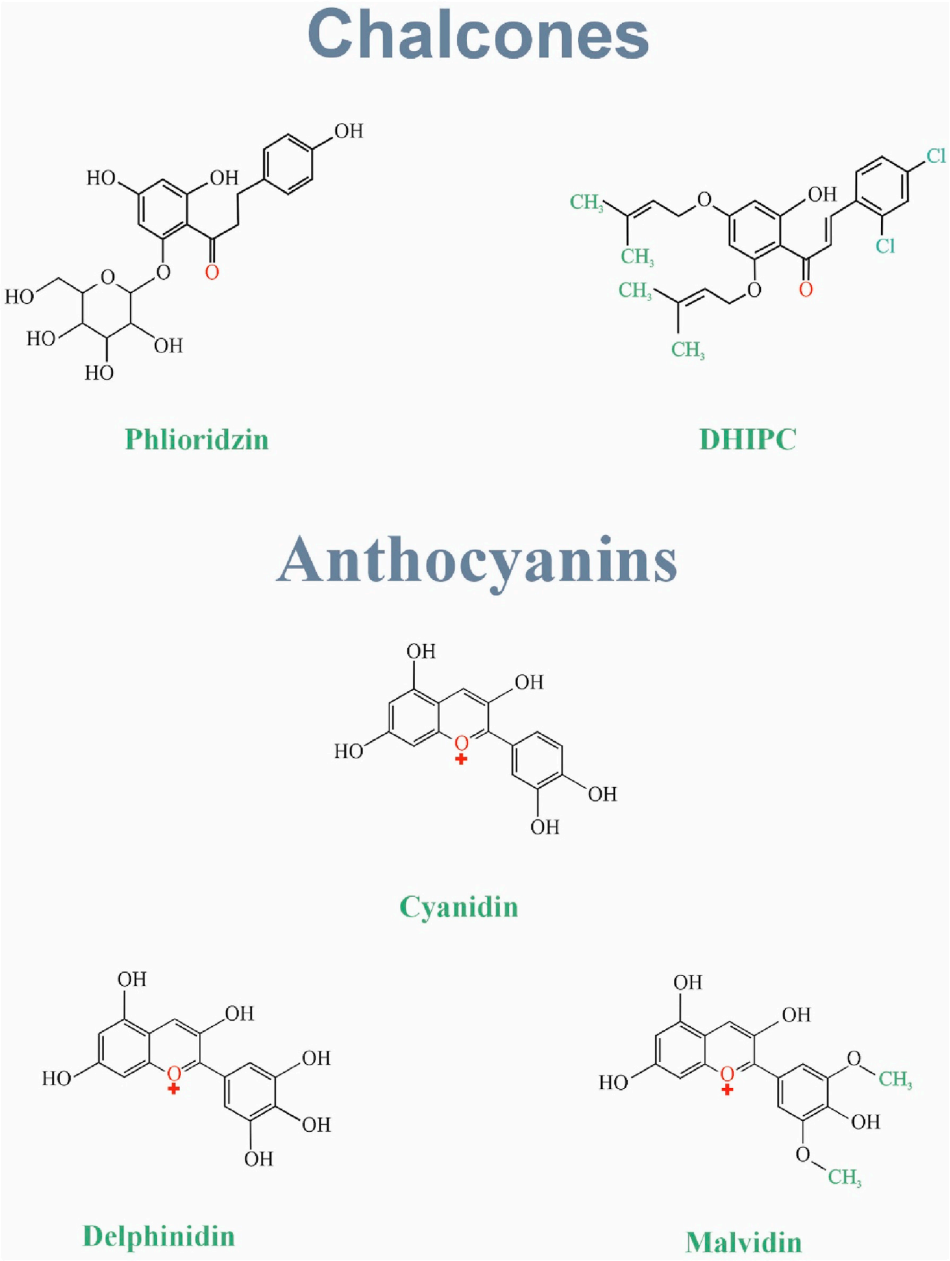
The structure of chalcones and anthocyanins family.

Chalcones were known to have an essential role in floral coloration and pollination by insects through their extremely conjoined structures (Rani et al., 2019). The main chalcone types are Phloridzin and Chalconaringenin (Jin, 2019). Chalcones make a significant class of natural compounds and flavonoid precursors plentiful in fruits, vegetables, and eatable plants (Panche, Diwan, and Chandra, 2016a). They are abundant in plants such as *Angelica keiskei*, *Glycyrrhiza inflates*, and *Piper aduncum* and have been consumed widely for their possible medical effects (Rani et al., 2019). Chalcones have properties against inflammation, oxidation, cancer, diabetes, and obesity (Panche, Diwan, and Chandra, 2016c). *In vivo* studies suggest they might even influence mood through interaction with serotonin receptors (Higgs et al., 2019). Further research is needed to explore their full potential, particularly regarding their possible role in treating depression. Figure 1 Shows the structure of chalcones family.

### 3.1 Phlioridzin

Apple peels, rich in phloridzin (Dhalaria et al., 2020), might offer a novel approach to managing depression. A study by (Kamdi, Raval, and Nakhate, 2021) suggests phloridzin improves mobility in diabetic mice, a behavioral indicator of reduced depression symptoms. Notably, phloridzin treatment also reversed the decrease in key biochemical markers associated with depression (GSH, BDNF, TrkB, CREB, ERK). These findings warrant further investigation into phloridzin’s potential as a therapeutic strategy for depression, particularly in diabetic patients.

### 3.2 Others

A new study by Zhao et al. suggests DHIPC (2,4-dichloro-2′-hydroxyl-4′,6′- diisoprenyloxychalcone), a synthetic chalcone compound, may hold promise for treating depression (Starchenko et al., 2020). In mice, oral DHIPC significantly reduced immobility time in behavioral tests, suggesting antidepressant effects. This improvement coincided with increased levels of key neurotransmitters (serotonin, noradrenaline, and 5-HIAA) in brain regions associated with mood regulation. (Zhao et al., 2018). These findings warrant further investigation of DHIPC’s potential as a therapeutic option for depression.

## 4 Anthocyanins

Anthocyanins, known as phenolic compounds with two benzene rings, are one of the flavonoid subclasses (Yousuf et al., 2016). More than 300 anthocyanins with various structures are found in nature. Cherries, strawberries, merlot grapes, raspberries, cranberries, blueberries, bilberries, and blackberries are among the natural sources of anthocyanins (Woo and Kim, 2013; Choy et al., 2019). Cyanidin, delphinidin, pelargonidin, petunidin, and malvidin are the most prevalent anthocyanins (Yousuf et al., 2016). Hydroxyl methylation or acylation of the A and B rings and pH is responsible for the color of anthocyanins (Panche, Diwan, and Chandra, 2016a). Today, their potential health factors as dietary antioxidants lead them to be used in preventing cancers, diabetes, and neuronal and cardiovascular diseases (Yousuf et al., 2016). Figure 1 Shows the structure of anthocyanins family.

### 4.1 Cyanidin

Preclinical studies suggest that dietary sources rich in anthocyanins, such as purple cauliflower, pomegranate, and blueberry, may possess antidepressant properties in mice. These studies demonstrate that cyanidin extracts can elevate levels of monoamine neurotransmitters (NE, DA, and 5-HT) through various mechanisms, including inhibition of monoamine oxidase (MAO) activity and increased brain-derived neurotrophic factor (BDNF) production. These effects ultimately contribute to neurogenesis and dendritic growth in the hippocampus, potentially leading to reversal of depressive behaviors. (Fang et al., 2020). *Punica granatum* methanolic pulp and peel extracts, which are rich in anthocyanins including cyanidin, delphinidin, and malvidin, were administered in male mice at doses of 25 and 50 mg/kg along with fluoxetine as the standard drug (20 mg/kg), respectively. This drug was introduced as a suitable antidepressant by reducing the immobility time in the forced swimming test (Abdul rahman et al., 2015). Blueberry contains various compounds such as Quercetin (QE), malvidin, and anthocyanidin. Gapski et al. (2019) found that blueberry extract could treat neurodegenerative diseases by modulating oxidative stress in the hippocampus. Administration of blueberry extract with doses higher than 300 mg/kg reduced the immobility time of mice in the tail suspension test (TST). Additionally, research suggests that cyanidin, a specific anthocyanin, may exert its antidepressant effects through the PI3K/AKT/FoxG1/FGF-2 pathway, which plays a critical role in neurogenesis (Shan et al., 2020). Other reported changes that attributed to the improved behaviors were restoration of glial fibrillary acidic protein (GFAP), brain-derived neurotrophic factor (BDNF), and glutamate-aspartate transporter (GLAST) and excitatory amino acid transporter 2 (EAAT2) expression (Qu et al., 2022). These findings collectively support the exploration of anthocyanin-rich dietary interventions as a potential strategy for managing depression.

### 4.2 Delphinidin

The effect of maqui berry extract (MBE) (25,50, and 100 mg/kg) containing cyanidin and delphinidin was investigated on post-stroke depressed male mice for 7 days. Treatment with MBE mitigated anhedonia and immobility time observed in mice. Interestingly, MBE led to a reduction of high levels of TBARS as well as an increase of antioxidant enzymes (Abdul rahman et al., 2015). Therefore, modulating oxidative stress could restore normal behavior in mice (Di Lorenzo et al., 2019).

### 4.3 Malvidin

Malvidin is an anthocyanin obtained from red wine that is proven to protect against oxidative neuronal damage in both animal cell lines and *in vivo* models (Khoo et al., 2017). Administration of malvidin-3′-*O*-glucoside was found to attenuate the depressive-like behavior through its ability to cross the BBB and upregulation of Rac1 expression in the nucleus accumbens (Wang et al., 2018), a protein whose depletion contributes to the disturbed synaptic structure in depression (Golden et al., 2013).

## 5 Flavonols

**FIGURE 2:**
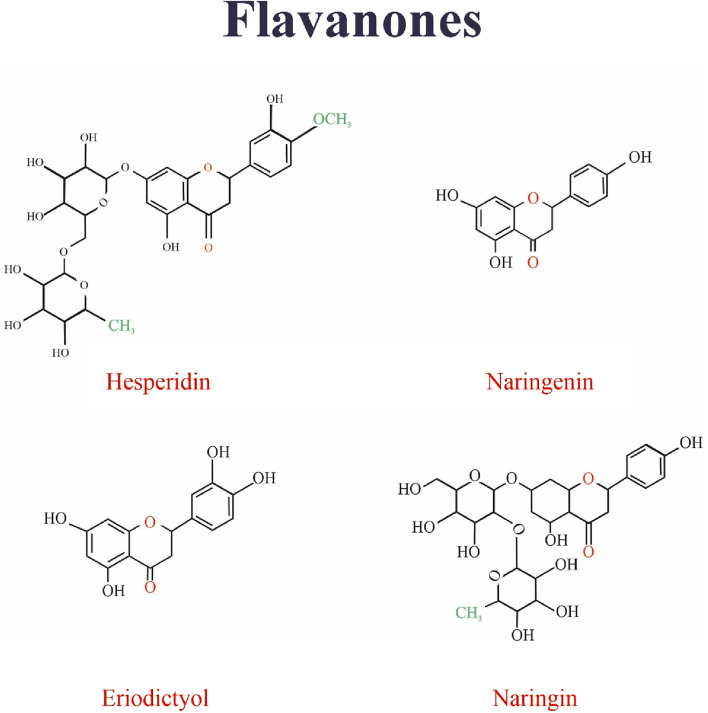
The structure of isoflavonoids family.

Flavonols are a subset of flavonoids with a group of ketones (Panche, Diwan, and Chandra, 2016b). The structural characteristic of flavonols is an unsaturated carbon ring at carbon 3-4, which is hydroxylated at Carbon 3 and oxidized at Carbon 4 (Al-Ishaq et al., 2019). Flavonols have a wide range of health benefits, such as reducing the risk of vascular diseases and antioxidant potential. Flavonols are abundant in fruits and vegetables (Panche, Diwan, and Chandra, 2016b). The studied flavonols include kaempferol (KFL), myricetin, quercetin, rutin, fisetin, silymarin, and isorhamnetin (Choy et al., 2019). Figure 2 Shows the structure of flavonoles family.

### 5.1 Rutin

**FIGURE 3:**
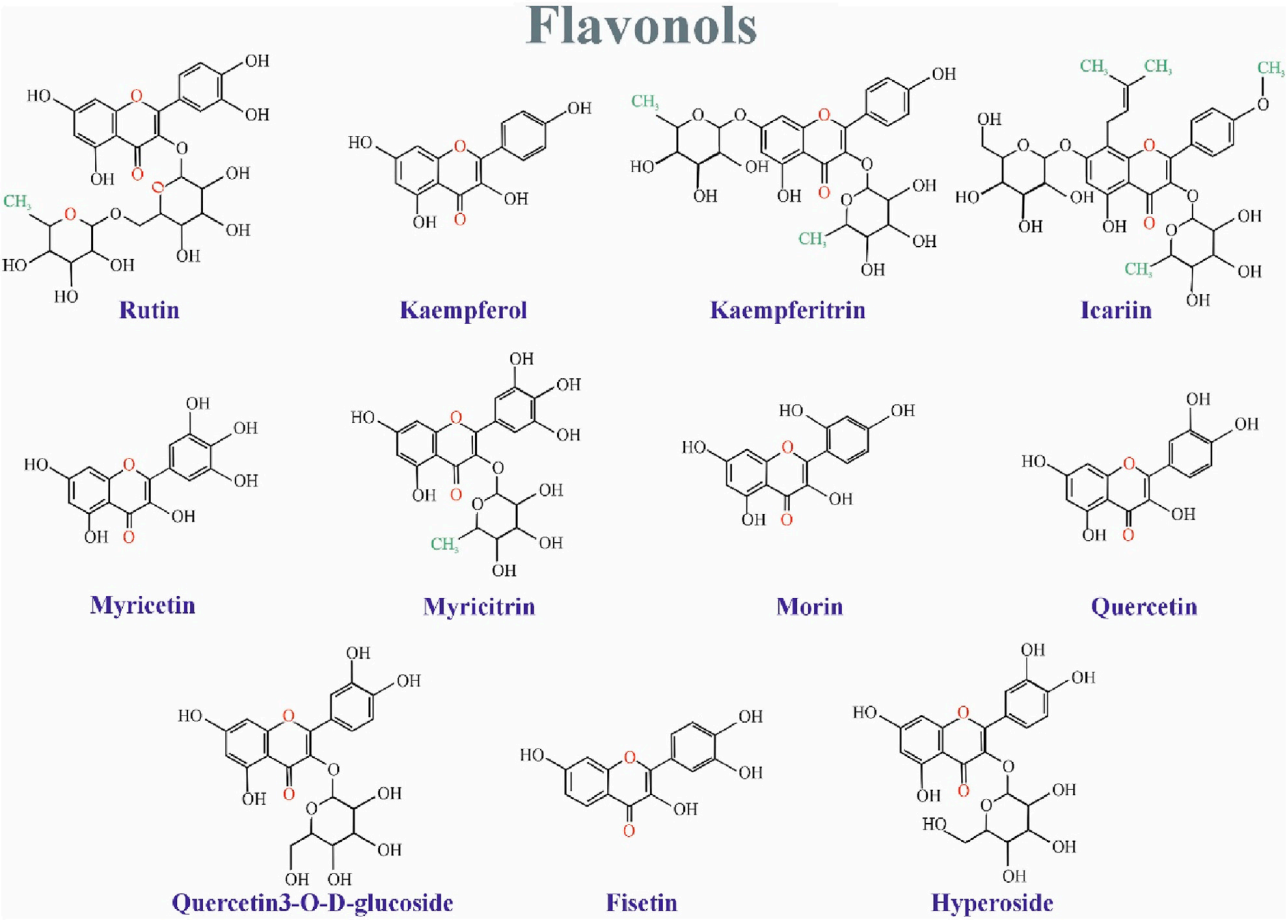
The Mechanistic Effect of Rutin, Quercetin, Cyanidin, Morin, Philoridzin, Malvidine, Myricetin, and Kaempferol on Depression. Rutin and quercetin can increase serotonin (5-HT). However, cyanidin and quercetin decrease monoamine oxidase (MAO). Rutin and quercetin can raise norepinephrine. Moreover, morin and quercetin can reduce pro-inflammatory cytokines secretion. Philoridzin, and cyanidin have positive effects on tropomyosin receptor kinase B (TrkB). kaempferol has a promising impact on protein kinase B (AKT), which can regulate BDNF translocation. Also, myricetin, and quercetin positively impact BDNF translocation. Finally, philoridzin, cyanidin, malvidine, myricetin, and quercetin have antioxidant effects.

Rutin, a citrus bioflavonoid, has emerged as a potential candidate for treating depression based on preclinical studies. (Pandey and Rizvi, 2009). Machado et al. demonstrated that the antidepressant-like effect of an ethanolic extract from Schinus molle, containing rutin, is likely mediated by increased synaptic availability of noradrenaline and serotonin. This finding suggests a potential mechanism for rutin’s action. (Machado et al., 2008). Yusha’u et al. (2017) further supported rutin’s antidepressant potential by observing a dose-dependent decrease in immobility time during the tail suspension test (TST) in mice treated with rutin (Yusha’u et al., 2017) (Figure 3). However, Scheggi et al. reported an inverted U-shaped dose response for rutin’s anti-stress effects, suggesting the existence of an optimal dosage for its efficacy (Scheggi et al., 2016). Similarly, Nöldner and Schötz (2002) observed a threshold effect for rutin’s contribution to the antidepressant properties of *Hypericum perforatum* extracts.

In a study by (Sommer and Harrer, 1994) one hundred and five outpatients with mild depression for a short period were administered either 3 × 300 mg hypericum extract or a placebo in 4 weeks. In the active group, 28 of 42 patients (67%) and, in the placebo group, 13 of 47 patients (28%) responded to treatment. The medicinal suitability of an antidepressant is contingent not only on its antidepressant-like effect but also on the essence and extent of its complications.

These studies highlight the potential of rutin for treating depression. Further research is necessary to elucidate the precise mechanisms of action and to establish a safe and tolerable dosing regimen. Additionally, exploration of potential side effects and interactions with other medications is crucial for responsible clinical development.

### 5.2 Kaempferol

Kaempferol, a 3, 4, 5, 7-tetrahydroxyflavone, is a natural compound found in a variety of sources including tea, broccoli, tomatoes, Ginkgo biloba L., grapes, and other fruits and vegetables (Al-Ishaq et al., 2019). Preclinical studies suggest antidepressant potential for kaempferol. Reduced immobility time in animal models treated with kaempferol support this possibility (Park et al., 2010). Gao et al. (2019) further demonstrated kaempferol’s antidepressive-like effects in a chronic stress model, potentially mediated by enhanced anti-inflammatory and antioxidant actions via upregulation of AKT/β-catenin cascade activity. These findings are promising, but further investigation into kaempferol’s structure-activity relationships (SAR) is crucial to optimize its efficacy for depression treatment. Additionally, research is needed to elucidate the specific mechanisms of action and potential advantages of different kaempferol derivatives. In a study by Yan et al. (2015), kaempferol-3-O-D-glucoside, kaempferol, quercetin, and quercetin-3-O-D-glucoside were purified from the hydroethanolic extract of Apocynum venetum L. leaves. The behavioral test indicated amelioration of depressive-like symptoms in mice. The further biochemical assessment showed that anti-depressant activity could be dependent on the increase of main neurotransmitter levels including serotonin, dopamine, and norepinephrine as well as a reduction in the rate of serotonin metabolism. Future studies should also explore kaempferol’s safety and tolerability in humans and its potential interactions with other medications. Ultimately, clinical trials are necessary to confirm kaempferol’s efficacy in treating depression.

### 5.3 Kaempferitrin

Kaempferitrin is a kaempferol dirhamnoside in which the hydroxyl groups at positions 3 and 7 are substituted. This compound is obtained from a variety of plants including *the Justicia spicigera* (Asteraceae) plant. Treatment with kaempferitrin is found to improve the performance of depressed mice in FST and TST. At the molecular level, kaempferitrin exerts its anti-depressant potential by modulating the activity of the serotonergic system, particularly through presynaptic 5-HT1A receptors and regulating hypothalamic-hypophysis-adrenal axis (HPA) (Cassani et al., 2014).

### 5.4 Icariin

Icariin, also known as a prenylated flavonol glycoside, is an 8-prenyl derivative of kaempferol 3,7-O-glucoside. It is predominantly found in *Herba epimedii*, a traditional Chinese herb that has been used for centuries to treat various ailments (Pannu A. et al., 2021). Animal models demonstrate its ability to prevent social aversion and alleviate depressive symptoms through diverse mechanisms, including restoring hypothalamic-pituitary-adrenal (HPA) axis function, enhancing brain-derived neurotrophic factor (BDNF) levels, and reducing neuroinflammation and nitric oxide production (Wu et al., 2013; Liu et al., 2015). Additionally, studies have revealed that icariin and associated improvements in behavioral tests are accompanied by anti-inflammatory action, anti-oxidant action, Inhibited activation of NF-kB signaling, as well as a decrease in levels of SGK1 (serum and glucocorticoid-regulated kinase 1) and FKBP5 (FK506 binding protein 5) expression (Wei et al., 2016). Notably, Di et al. (2023) observed increased expression of monoamine neurotransmitters and BDNF-TrkB signaling pathway genes in mice treated with icariin, further supporting its potential antidepressant action (Di et al., 2023). While these findings are promising, investigations into SAR are essential to optimize icariin’s efficacy for depression treatment. Clinical trials are ultimately necessary to confirm its safety and efficacy in humans. Furthermore, icariin’s multi-target approach warrants further exploration to elucidate the relative contributions of each mechanism and potentially reveal advantages over traditional antidepressants.

### 5.5 Myricetin and myricitrin

Myricetin is a hexahydroxyflavone and is rich in fruits, vegetables, nuts, red wine, berries, and tea (Taheri et al., 2020). In a study by Ma et al. (2015) TST results showed that myricetin decreased immobility time. Biochemical assessments showed myricetin elevated the activities of glutathione peroxidase (GSH-PX) in the hippocampus of mice. In addition, it reduced plasma corticosterone levels of those mice to repeated restraint stress. In one study, it was shown that repeated treatment with myricitrin at 10 mg/kg resulted in increased mobility time in TST, which is possibly mediated by myricitrin-induced hippocampal neurogenesis and inhibition of nitric oxide (Meyer et al., 2017).

### 5.6 Morin

Preclinical studies suggest antidepressant potential for morin, a naturally occurring flavonoid. In a chronic stress model, morin treatment improved levels of key neurotransmitters (epinephrine, norepinephrine, and 5-HT) and reduced inflammatory markers including tumor necrosis factor-alpha, toll-like receptor-4, NOD-like receptor pyrin domain-containing protein-3, interleukin-1beta, and caspase-1 levels, as well as the status of the critical apoptotic marker, caspase-3 in the brain. These findings suggest its potential to alleviate depression through neurochemical and anti-inflammatory pathways (Hassan et al., 2020). However, Ben-Azu et al. (2019) observed that L-arginine, a substrate for nitric oxide synthesis, reversed morin’s antidepressant effects in mice. This suggests the involvement of the N(G)-nitro-l-arginine methyl ester is L-NAME in morin’s mechanism of action.

**FIGURE 4:**
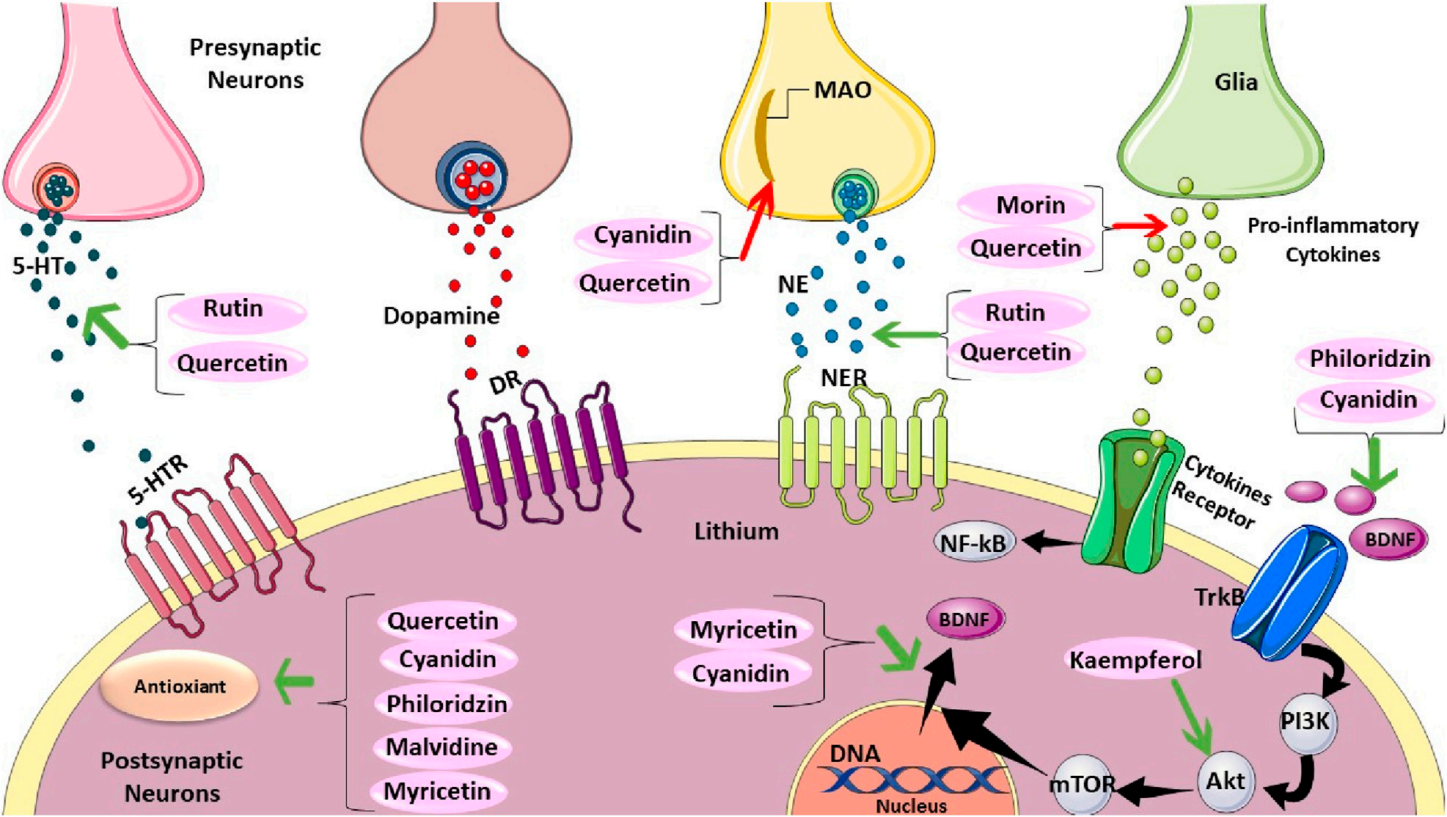
The Mechanistics Effect of Apigenin, Biacalein, Genistein, Hesperidin, and Narnigenin. Genistein, baicalein, hesperidin, and naringenin can increase serotonin (5-HT). Also, apigenin and biacalein can elevate dopamine. However, genistein can decrease monoamine oxidase. Moreover, apigenin also can reduce pro-inflammatory cytokines secretion. Apigenin and naringenin can raise norepinephrine. Furthermore, Hesperidin positively affects TrkB. Apigenin has a promising impact on mTOR, which can regulate BDNF translocation. Hesperidin has adverse effects on NF-kB. Baicalein, naringenin, and hesperidin have positive effects on BDNF translocation. Finally, apigenin, baicalein, and naringenin have antioxidant effects.

Quercetin is a pentahydroxyflavone and highly abundant in apples, onions, broccoli, wine, and plants such as green tea and *Ginkgo biloba*. Quercetin or its derivatives have been shown to alleviate depressive behaviors, potentially through various mechanisms including regulating neurotransmitter levels (Şahin et al., 2019; Samad et al., 2018; Yan et al., 2015), reducing oxidative stress (Şahin et al., 2019; Scheggi et al., 2016), microglia activation, and stress-induced apoptosis (Rinwa and Kumar 2013; Park et al., 2010). Additionally, the anti-depressant effect of quercetin is suggested to be possible via restoring serotonin levels and preventing brain oxidative stress by inhibiting MAO-A activity (Singh, Chauhan, and Shri, 2021) (Figure 4). Moreover, Hou et al. (2010) revealed that quercetin modulated depression-related signaling pathways involving BDNF and phosphorylation of cyclic adenosine monophosphate (AMP) response element-binding protein (CREB) and decreased amyloid-b peptide (Ab) in neurons isolated from double transgenic AD mouse. were reported.

Quercetin’s multifaceted effects on depression, potentially including memory enhancement, warrant further exploration to elucidate distinct mechanisms and potential advantages over traditional antidepressants.

Quercetin3-O- -D-glucoside is a type of quercetin O-glucoside. In this compound, quercetin is substituted with a -D-glucosyl residue at position C3. Among four flavonoids that were isolated from Apocynum venetum leaf, quercetin3-O-D-glucoside also showed anti-depressive activity with both gross changes (reduced immobility time) and biochemical alterations such as increased NE, DA, and 5-HT and reduced 5-HT metabolism (Yan et al., 2015).

### 5.7 Fisetin

Fisetin is a bioactive flavonoid found plentifully in vegetables and fruits, particularly in strawberries (Shukla et al., 2019). It demonstrates dose-dependent antidepressant effects in animal models, reducing immobility time and increasing serotonin and noradrenaline levels (Zhen et al., 2012). Fisetin’s potential mechanisms include anti-inflammatory activity, activation of the TrkB signaling pathway, and downregulation of the TNF-α/NLRP3 inflammasome (Yu et al., 2016; Wang et al., 2017; Gopnar et al., 2023). While fisetin demonstrates promise as a natural antidepressant, its synergistic potential with other natural compounds or traditional antidepressants warrants investigation. Exploring combinations could potentially enhance efficacy or address limitations associated with single-compound therapy.

### 5.8 Hyperoside

Hyperoside, a natural flavonol, is commonly referred to as 3-O-galactoside of quercetin due to the presence of a β-D-galactosyl residue attached to C3. Multiple mechanisms have been proposed to explain its antidepressant effects. Haas et al. (2011) suggest hyperoside activates D2 dopamine receptors, potentially contributing to its efficacy. Additionally, studies by Zheng et al. (2012) and (Butterweck, Hegger, and Winterhoff 2004) indicate hyperoside may influence neurotrophic factors (BDNF, CREB) via the AC-cAMP-CREB pathway and regulate the hypothalamic-pituitary-adrenal (HPA), respetively. Recently, Song et al. (2022) reported the same symptom-relieving effect of hyperoside on CUMS challenged mice. Further assessment revealed that hyperoside could exert an anti-depressant effect by inhibiting the NLRP1 inflammasome through the CXCL1/CXCR2/BDNF signaling pathway. Compared to traditional antidepressants targeting specific neurotransmitters, hyperoside appears to act through multiple mechanisms, potentially offering a broader therapeutic approach for depression. Further research elucidating the relative contributions of each mechanism could reveal hyperoside’s potential for addressing different subtypes of depression or reducing side effects associated with single-target drugs.

## 6 Isoflavonoids

Isoflavones comprise a very distinct and significant subgroup of flavonoids (Panche, Diwan, and Chandra, 2016b). They are also a group of phytoestrogens that include daidzein, glycitein, genistein, biochanin A, irilone, and formononetin (Wu, Sang, and Chang, 2020). Isoflavones can be found mostly in soybeans, legumes, and some microbes (Borges Filho et al., 2016a; Al-Ishaq et al., 2019). Genistein, daidzein, and glycitein are found in soybeans, formononetin, biochanin A, and irilone in clover (Wu, Sang, and Chang, 2020). Figure 2 Shows the structure of Isoflavonoids family.

### 6.1 Genistein

**FIGURE 5:**
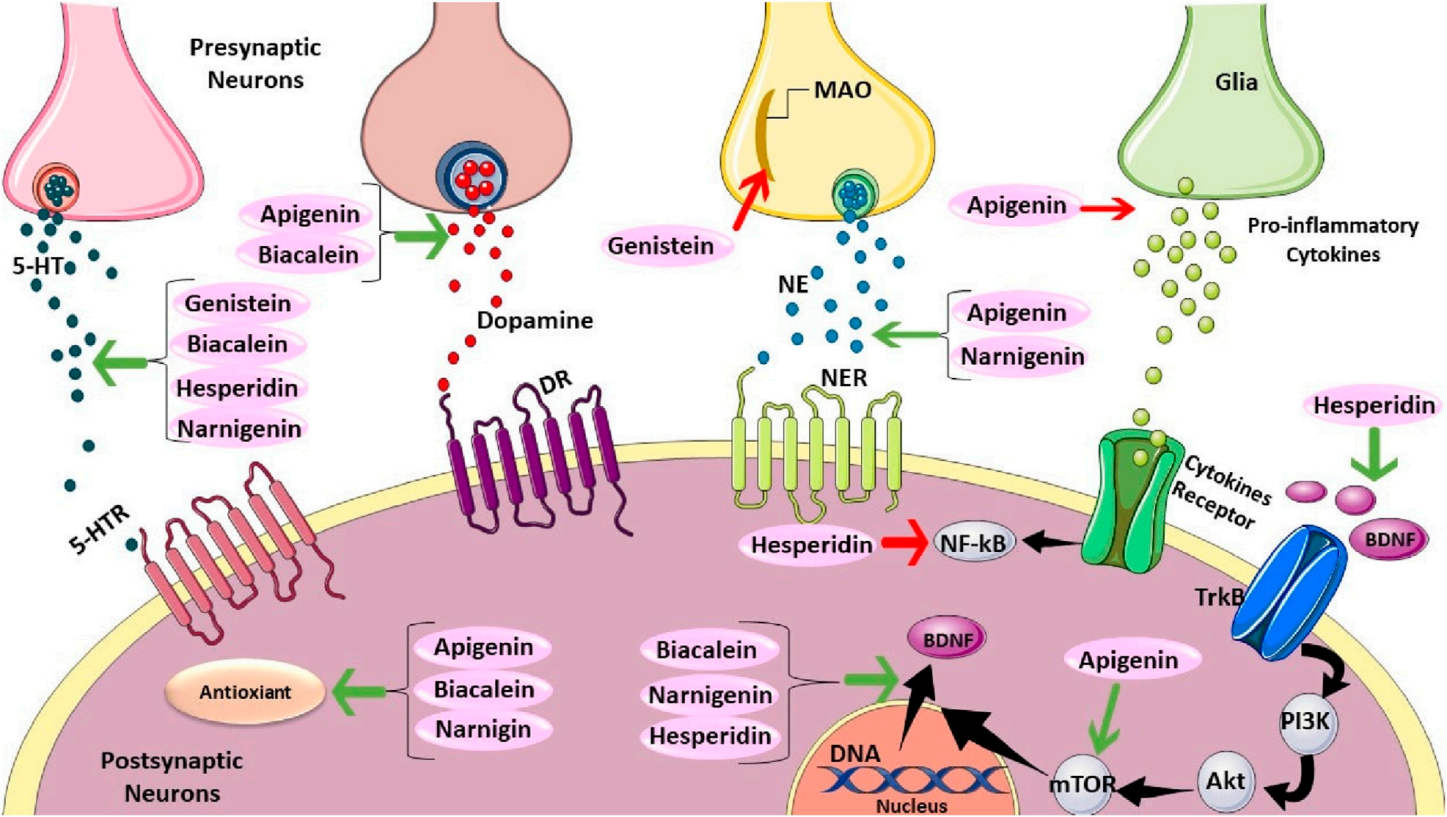
The structure of flavonols family.

Genistein is an isoflavonoid compound derived from soy that is known for its numerous health benefits (Setchell, Brown, and Lydeking-Olsen, 2002). It exhibits antidepressant-like effects through diverse mechanisms. Shen et al. (Shen et al., 2018) propose genistein downregulates miR-221/222, targeting connexin 43, a protein linked to depression. Furthermore, studies suggest genistein regulates monoamine metabolism, particularly serotonin, potentially explaining its antidepressant properties (Kageyama et al., 2010) (Figure 5).

Hu et al. (2017) observed a dose-dependent antidepressant effect in mice treated with genistein, accompanied by increased brain monoamines (noradrenaline, serotonin, and dopamine and their metabolites (5-HIAA and DOPAC)) and decreased monoamine oxidase activity. Another study investigated the acute and chronic antidepressant-like effects of 10 mg/kg of genistein in combination with 5 and 10 mg/kg of amitriptyline for 10 days on male albino mice. Notably, Gupta et al. (2015) demonstrated a synergistic effect when combining genistein with amitriptyline, suggesting potential benefits for treatment-resistant depressionAdditionally, a clinical study examining the effects of genistein consumption in postmenopausal women with osteopenia showed improved quality of life and alleviation of depression symptoms following the treatment (Atteritano et al., 2014). Genistein’s multi-target action, including microRNA modulation and neurotransmitter regulation, warrants further exploration to elucidate its potential advantages over traditional antidepressants, particularly in treatment-resistant cases and combination therapies.

### 6.2 Daidzein

Daidzein, an isoflavone commonly found in soy, can be converted into equol, a compound with higher estrogenic potency than other isoflavones (Setchell, Brown, and Lydeking-Olsen, 2002). Chen et al. (2021) showed that daidzein effectively reversed the inescapable shock-induced helplessness behavior in a group of learned helplessness rats, and the immobility time in CUMS challenged mice via mitigating HPA axis hyperactivity, and partially correcting the imbalances in inflammatory cytokines.

## 7 Flavones

**FIGURE 6:**
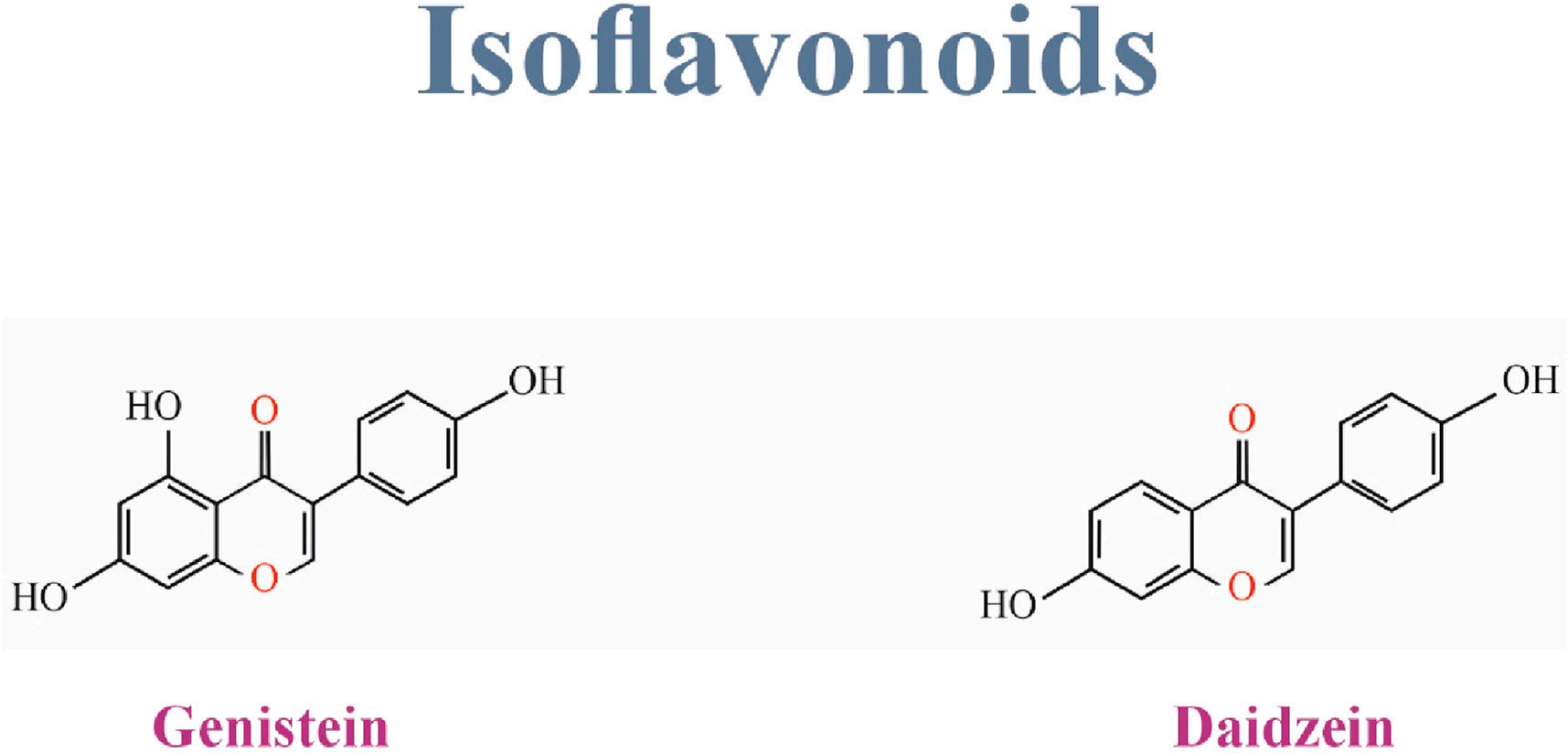
The structure of flavones family.

Mainly discussed flavones are luteolin, Apigenin, and tangerine (Choy et al., 2019). Flavones have a hydroxyl functional group in their A ring, a ketone, and a double bond in their C ring (Panche, Diwan, and Chandra, 2016a). They are found in flowers, fruits, and leaves such as red peppers, celery, parsley, chamomile, mint, and Ginkgo biloba. Luteolin, a common flavone, is mainly in vegetables and fruits such as carrots, cabbages, parsley, broccoli, celery, and apple peels. Apigenin is one of the most widespread flavones in grapefruits, oranges, celery, and onions (Choy et al., 2019). Figure 6 Shows the structure of Isoflavonoids family.

### 7.1 Apigenin

Apigenin is one of the most widespread flavones in grapefruits, oranges, celery, and onions. It demonstrates antidepressant-like effects through diverse mechanisms. Antidepressant outcome of apigenin might be related to the inhibition of pro-inflammatory cytokines (tumor necrosis factor-alpha (TNF-α) and interleukin-1β (IL-1β), inducible Nitric Oxide Synthase (iNOS), and cyclooxygenase-2 (COX-2) utterance in the brain (Li et al., 2015). Furthermore, apigenin appears to modulate neurotransmitter levels (dopamine, serotonin) (Nakazawa et al., 2003; Yi et al., 2008), and increase BDNF (Weng et al., 2016).

Additionally, 20 mg/kg apigenin for 3 weeks in male mice treated with CUMS caused a decrease in the levels of provocative brain cytokines (IL-18 and IL-1β), changes in oxidative stress parameters (reducing malondialdehyde (MDA) levels, and restoring the reduced glutathione (GSH) levels), and a decrease in expression of NLRP3 inflammatory activation (Li et al., 2016b). Moreover, apigenin treatment was found to exert anti-depressive effects by increasing the level of hippocampal microtubule-associated protein light chain 3-II/I (LC 3-II/I) while decreasing p62 expression, the levels of adenosine monophosphate-activated protein kinase (AMPK) and Unc-51 like autophagy activating kinase-1 (ULK1). It also inhibited the levels of mTOR. overall indicating that apigenin potentially increased autophagy through the AMPK/mTOR pathway (Zhang et al., 2019) (Figure 4). Apigenin’s multifaceted approach, targeting inflammation, neurotransmitters, BDNF, and autophagy, suggests potential advantages over traditional antidepressants with more limited mechanisms of action. Further research is warranted to elucidate the relative contributions of each mechanism and explore apigenin’s efficacy in human trials.

### 7.2 Baicalein

Baicalein demonstrates diverse mechanisms underlying its antidepressant-like effects. Studies suggest baicalein activates the ERK and BDNF pathways in the hippocampus, potentially promoting neurotrophic activity (Xiong et al., 2011). Additionally, it appears to alleviate neuroinflammation by inhibiting the NF-κB pathway (Du et al., 2019), restore dopamine levels and increase BDNF expression (Lee et al., 2013), and even enhance hippocampal neurogenesis through the cAMP/PKA pathway (Zhang et al., 2018). The superior potency of Baicalein compared to other flavones warrants further investigation. This suggests Baicalein’s potential as a lead compound for antidepressant development due to its multi-targeted mechanisms and potentially broader spectrum of action.

### 7.3 Chrysin

The flavone chrysin is contained in various plants and is also found in propolis and honey, and many studies have shown its antidepressant-like effects (Rao et al., 2016; Rodríguez-Landa et al., 2022). It can also affect different brain parts, such as the prefrontal cortex, hippocampus, and raphe nucleus (Rodríguez-Landa et al., 2022). It can activate neurological factors, regulate biomarkers of oxidative stress, and regulate the apoptotic signaling pathway, which causes the antidepressant-like effects of this flavonoid (Rodríguez-Landa et al., 2022).

Several studies have shed light on the diverse mechanisms underlying chrysin’s antidepressant potential. One mechanism involves modulation of the serotonergic system.German-Ponciano et al. (2021) demonstrated that chronic administration of chrysin in rats influenced the expression of 5-HT receptor subtypes in brain regions associated with mood, suggesting its ability to regulate serotonin signaling. Furthermore, chrysin appears to positively influence the production of neurotrophic factors like BDNF and NGF (Jesse et al., 2015). These neurotrophic factors play a crucial role in neuronal growth, survival, and differentiation, and their downregulation is implicated in depression. By promoting neurotrophic factor production, chrysin may contribute to neurogenesis and neuronal resilience, potentially reversing depressive symptoms.

Another promising aspect of chrysin’s antidepressant potential lies in its antioxidant and anti-inflammatory properties. Studies by Borges Filho et al. have shown that chrysin can counteract oxidative stress and inflammation (Borges Filho et al., 2016a). By mitigating these detrimental processes, chrysin may offer neuroprotective effects and alleviate depressive symptoms associated with neuroinflammation.

Chrysin’s interaction with the GABAergic system, another critical pathway involved in mood regulation, presents a further avenue for investigation. Cueto-Escobedo et al. (2020) observed that chrysin’s antidepressant-like effects were blocked by an antagonist of the GABAergic system, suggesting its potential role in modulating GABAergic neurotransmission (Cueto-Escobedo et al., 2020). Further research is necessary to elucidate the precise mechanisms by which chrysin interacts with this system.

Additionally, German-Ponciano et al. (Germán-Ponciano et al., 2020), compared the results of a single injection and 21 consecutive days of treatment with chrysin 2, 4, and 8 μmol kg^−1^ on anxiety-like behavior in male Wistar rats. The authors compared the effects of chrysin with anxiolytic diazepam 7 μmol kg^−1^. They indicated that diazepam had similar effects as acute (not chronic) treatment, with 4 μmol kg^−1^ chrysin exerting anxiolytic- and anti-depressant-like effects.

The multifaceted mechanisms of chrysin, targeting the serotonergic system, neurotrophic factors, inflammation, and the GABAergic system, suggest potential advantages over traditional antidepressants with more limited mechanisms of action (Borges Filho et al., 2016b). Further research is warranted to elucidate the relative contributions of each mechanism and explore chrysin’s efficacy in human trials. If successful, chrysin could offer a novel therapeutic approach for treating depression with a broader spectrum of action and potentially fewer side effects.

### 7.4 Luteolin

Luteolin is a 30, 40 5, 7, tetrahydroxyflavone natural flavonoid and also found to easily cross BBB. Luteolin administration in rats exposed to single prolonged stress who developed depressive-like behavior has resulted in alleviated depression- and anxiety-like behaviors and reversed the elevated plasma corticosterone and adrenocorticotropic hormone levels. These changes were accompanied by a decrease in norepinephrine and an increase in serotonin levels in the prefrontal cortex and hippocampus. Therefore, the anti-depressant properties of luteolin could be due to its role in correcting the HPA axis dysregulation (Sur and Lee, 2022). similarly Cheng et al. (2022) demonstrated that luteolin’s anti-depressant activity could be attributed to the reversal of neuroinflammation. In this study, luteolin treatment not only effectively reduced inflammation in the hippocampus and prefrontal cortex but also enhanced synapsin levels. Also, a significant increase in the levels of serum 5-hydroxytryptamine and norepinephrine was observed in the treatment group. Several other studies also provided evidence of luteolin’s function as an anti-depressant agent (Ishisaka et al., 2011; de la Peña et al., 2014). Luteolin’s multifaceted approach, targeting stress hormones, neuroinflammation, synaptogenesis, and monoamine neurotransmitters, suggests potential advantages over traditional antidepressants.

### 7.5 Nobiletin

Nobiletin is a safe and non-toxic flavonoid compound extracted from citrus peel. It belongs to the group of polymethoxylated flavonoids (Alam et al., 2022). According to Wang et al. (2020) nobiletin administration in rats exposed to LPS has been found to improve the behavioral patterns related to depression. The molecular assessment confirmed that nobiletin reversed the neuroinflammation caused by LPS, promoted autophagy, and suppressed the activation of NLRP3 inflammasome possibly via the AMPK pathway. In another study, nobiletin was also found to attenuate depressive behavior according to TST and FST results. In this study, the anti-depressant potential of nobiletin was ascribed to its interaction with noradrenergic, serotonergic, and dopaminergic systems (Yi et al., 2011).

### 7.6 7, 8-dihydroxyflavone

7,8-dihydroxyflavone is a natural flavone that has demonstrated antidepressant effects in various studies. This flavonoid can penetrate the blood-brain barrier and is suitable for oral consumption, making it capable of interacting with critical pathways involved in depression in the brain (Yang and Zhu, 2022). The antidepressant effects of 7,8-dihydroxyflavone are primarily reported to be associated with increased expression of BDNF. Two studies have shown that chronic administration of 7,8-dihydroxyflavone improves depression-like behaviors in rats by acting as an agonist on the TrkB receptor, which subsequently increases hippocampal expression of BDNF (Zhang et al., 2014; Zhang et al., 2016). In addition, an additive effect of 7,8-dihydroxyflavone on conventional depression treatments, such as fluoxetine, has been observed to improve behavioral performance tests (Amin et al., 2020). This effect was associated with the upregulation of autophagy (a process that maintains energy homeostasis) through increased activation of the PI3/Akt/mTOR/ERK signaling pathway.

### 7.7 Amentoflavone

Amentoflavone is a biflavonoid compound consisting of a dimer of apigenin at position C8. It possesses a range of pharmacological properties, including neuroprotective, antioxidative, and anti-inflammatory effects (Yu et al., 2017). Additionally, there is evidence to suggest that it can cross the blood-brain barrier. In a preclinical study, Ishola et al. (2012) found that amentoflavone displayed anxiolytic and antidepressant effects in mice mediated by interactions with 5-HT2 receptors, α1-and α2-adrenoceptors, while the anxiolytic effect appears to involve the GABAergic system. Overall, these findings suggest that amentoflavone may exert antidepressant effects by modifying neurotransmitters and their receptors.

## 8 Flavanones

**FIGURE 7:**
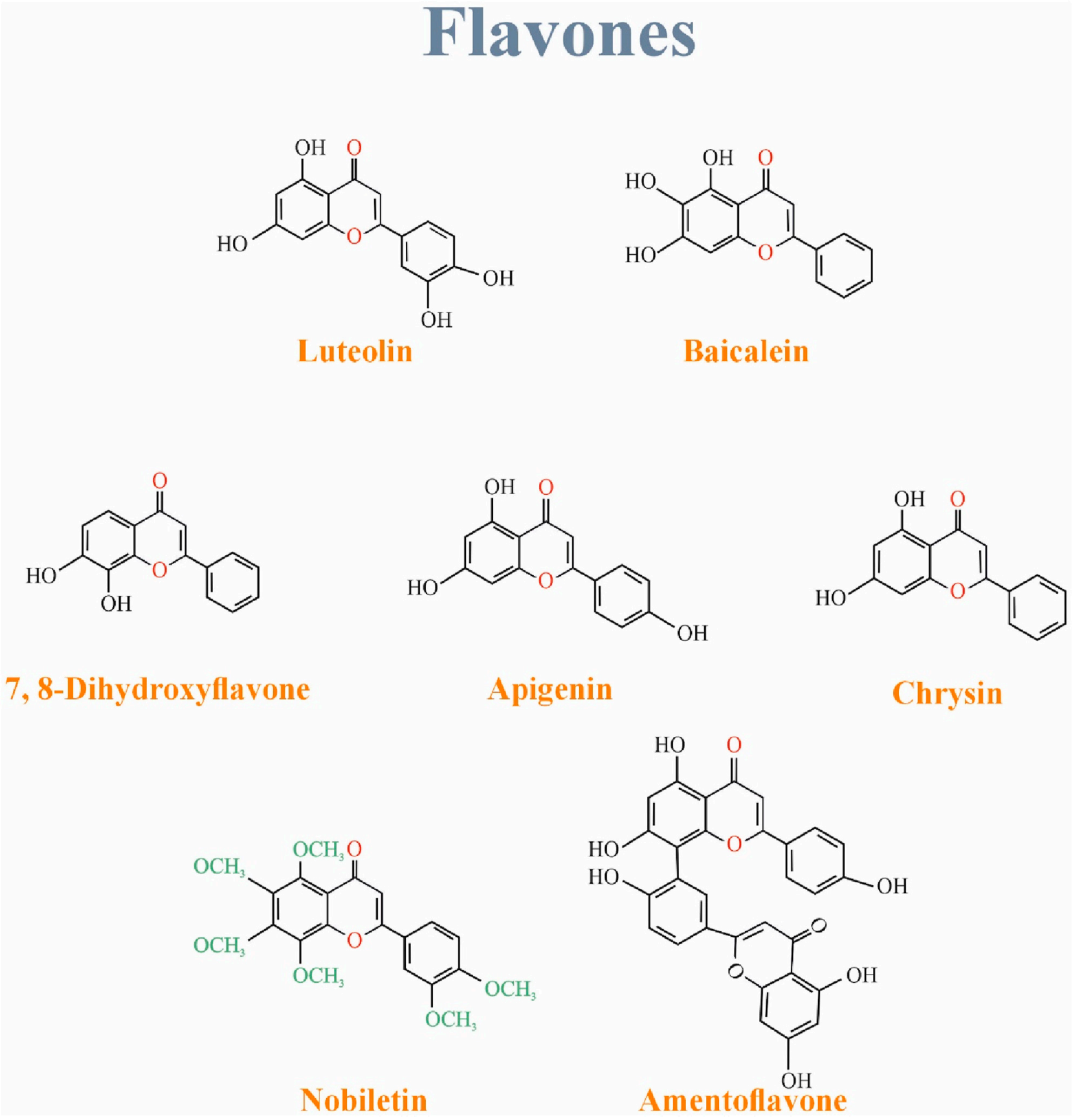
The structure of flavanones family.

Figure 7 Shows the structure of flavanones family.

### 8.1 Hesperidin

Hesperidin (4′-methoxy-7-O-rutinosyl-3′,5-dihydroxyflavanone) is a natural bioflavonoid primarily found in citrus fruits such as fingered citron (Garg et al., 2001).

Multiple studies have shed light on the diverse mechanisms underlying hesperidin’s antidepressant potential. One mechanism involves modulating inflammatory pathways. Fu et al. (2019) demonstrated that hesperidin treatment in mice exposed to chronic unpredictable stress reduced levels of pro-inflammatory cytokines (IL-6, IL-1β, and TNF-α). This anti-inflammatory effect appears to be mediated by the attenuation of the BDNF/TrkB and high-mobility–group box; chromosomal protein 1 (HMGB1)/receptor for advanced glycation end-product (RAGE)/NF-κB signaling pathways, both of which are implicated in depression. Furthermore, hesperidin appears to promote neurotrophic activity by increasing BDNF expression through various mechanisms (Donato et al., 2014; Li et al., 2016a).

In another study, Donato et al. (2015) also showed that hesperidin could play its antidepressant role by the inhibition of K+ channels, leading to the inhibition of the L-NAME pathway in male mice.

Hesperidin’s influence extends to neurotransmitter systems. Studies suggest it may modulate serotonin and dopamine levels in the brain (Souza et al., 2013; Nadar et al., 2018). Hesperidin’s ability to influence these neurotransmitters suggests a potential avenue for its therapeutic effects. Additionally, hesperidin exhibits antioxidant properties. A study by Filho et al. (2013) indicated that hesperidin could play its antidepressant role in the FST by the interaction with the k-opioid receptors in male mice. Diabetic male rat models treated with had enhanced Glo-1, an enzyme that detoxifies alpha-carbonyl aldehydes. Activation of the nuclear factor erythroid 2-related factor 2 (Nrf2)/antioxidant response element (ARE) pathway is known to be responsible for this change. Therefore, administering hesperidin might play an antidepressant-like role in diabetic rats (Zhu et al., 2020). Animal models of posttraumatic stress disorder (PTSD) treated with hesperidin (20, 50, and 100 mg/kg) for 14 days had a decreased 5-HIAA/5-HT ratio (Lee Bombi et al., 2020). *Postartum* depression mouse models treated with hesperidin (0.1, 0.5, and 1 mg/kg) showed decreased immobility time in TST and FST in *postpartum* mice. Molecular examination revealed a decrease in MDA levels after hesperidin treatment, suggesting an antioxidative mechanism that may contribute to the compound’s potential antidepressant effects. The results show that treatment with hesperidin can be effective (Khodadadeh, Hassanpour, and Akbari, 2020). Several additional studies have demonstrated the alleviation of depressive behavior in animal models of depression treated with hesperidin. These studies proposed various mechanisms underlying its effects, including increased neurogenesis and BDNF levels, increased ERK phosphorylation (Li et al., 2016a), modulation of pro-inflammatory cytokines (Antunes et al., 2016; Xie et al., 2020), suppression of microglia activation (Xie et al., 2020), and inhibition of acetylcholinesterase activity (Antunes et al., 2016). Hesperidin’s multifaceted approach, targeting inflammation, neurotrophic factors, neurotransmitters, and oxidative stress, suggests potential advantages over traditional antidepressants with more limited mechanisms of action.

### 8.2 Naringenin

Naringenin, a trihydroxyflavanone, is abundant in the peel of citrus fruits (Amin N. et al., 2020).

Multiple studies have shed light on the diverse mechanisms underlying naringenin’s antidepressant potential. One mechanism involves modulating the kynurenine pathway, a metabolic pathway of the amino acid tryptophan. Dysregulation of this pathway, leading to increased production of neurotoxic metabolites, is implicated in depression (Bansal et al., 2018). Naringenin treatment in animal models has been shown to restore imbalances in the kynurenine pathway, potentially mitigating its detrimental effects. Furthermore, naringenin exhibits anti-inflammatory properties, reducing levels of pro-inflammatory cytokines like TNF-α, IL-6, and IL-1β (Bansal et al., 2018). Chronic inflammation is increasingly recognized as a contributing factor in depression, and naringenin’s ability to counteract this process may contribute to its therapeutic effects.

Naringenin’s influence extends beyond inflammation and neurotransmitter modulation. Studies suggest it may activate the Sonic Hedgehog (SHH) signaling pathway (Tayyab et al., 2019). In animal models of depression, naringenin treatment has been shown to upregulate BDNF, SHH, GLI1, NK2 Homeobox 2 (NKX2.2), and Paired box protein Pax-6 (PAX6). Additionally, naringenin appears to enhance serotonergic and noradrenergic systems by increasing levels of serotonin, norepinephrine, and glucocorticoid receptors in the hippocampus (Yi et al., 2010). These neurotransmitters play a key role in mood regulation, and their dysregulation is associated with depression. Naringenin’s ability to influence these systems suggests a potential mechanism for its antidepressant effects.

Investigating the underlying mechanisms involved in the anti-depressant properties of naringenin revealed the involvement of the BDNF signaling pathway. Naringenin was found to increase the expression of BDNF in the hippocampus of depressed rats in several studies, resulting in improved outcomes of behavioral tests (Yi et al., 2014; Zhang et al., 2023; Olugbemide et al., 2021). Several studies have also highlighted naringenin’s antioxidant and anti-inflammatory properties (She et al., 2021). For instance, studies have shown that naringenin treatment reduces oxidative damage, decreases the expression of the pro-inflammatory NF-κB protein, and shifts the microglia population towards the anti-inflammatory M2 phenotype (Zhang et al., 2023). These findings suggest that naringenin may target multiple aspects of the neuroinflammatory response, potentially contributing to its antidepressant effects.

### 8.3 Naringin

Naringin, a flavanone-7-O-glycoside compound consisting of 40-hydroxyflavanones, is naturally present in citrus fruits, with grapes exhibiting significantly higher concentrations. This organic compound is responsible for the bitter taste of fruits (Suseem and Dhanish, 2019). Naringin exhibits a surprising benefit–antidepressant- and anxiolytic-like effects in mice. Behavioral improvements across various tests, such as reduced immobility time and increased social interaction preference, suggest naringin’s potential therapeutic value (Ben-Azu et al., 2018).

Studies suggest naringin exerts neuroprotective effects by reducing oxidative stress and improving mitochondrial function in models of post-stroke depression (Aggarwal, Gaur, and Kumar 2010). Oxidative stress is increasingly recognized as a contributing factor in depression, and naringin’s ability to mitigate these detrimental processes may offer neuroprotective benefits and alleviate depressive symptoms associated with neuroinflammation. Furthermore, naringin appears to target specific signaling pathways in the hippocampus. Studies have shown that naringin can inhibit NMDA receptors, potentially regulating neuronal excitability, and promote neurogenesis via activation of the cAMP response element-binding protein (CREB) signaling pathway, which plays a crucial role in the growth and survival of new neurons (Wang et al., 2023; Gao et al., 2022). These findings suggest naringin may promote neuroplasticity, the brain’s ability to adapt and form new connections, potentially offering a long-term therapeutic benefit for depression.

Naringin’s influence may extend beyond neuroprotection and neurogenesis. Studies suggest it may modulate neurotransmitter systems by normalizing the activity of the enzyme AchE, thereby potentially influencing levels of the neurotransmitter acetylcholine (Oladapo et al., 2021). Additionally, naringin appears to promote GABAergic neurotransmission, potentially and reducing neuroinflammation as well as oxidative stress. Similarly, Kwatra et al. (2016) reported the same reduction in the levels of pro-inflammatory cytokines as well as a decrease in the previously elevated serum corticosterone. The authors also examined the synergistic effect of naringenin with sertraline in mice with depression and discovered that the combination of these two substances alleviated depressive-like behavior through the regulation of the 5-HT level and protection of the mitochondrial complexes pathway, as well as by reducing oxidative stress in the hippocampus. These findings suggest naringin may exert its antidepressant effects through a multifaceted approach, targeting multiple neurotransmitter systems. Moreover, naringin exhibits anti-inflammatory properties, as evidenced by reduced levels of pro-inflammatory cytokines. Chronic inflammation is increasingly recognized as a contributing factor in depression, and naringin’s ability to counteract this process may contribute to its therapeutic effects.

### 8.4 Eriodictyol

Eriodictyol is a flavanone widely abundant in medicinal plants, citrus fruits, and an array of vegetables (Islam et al., 2020). Zhang et al. (2020) assessed the antidepressant-like effects of chronic eriodictyol (10, 30, and 100 mg/kg) in male rat models of depression. The authors reported that 100 mg/kg eriodictyol for 28 days had antidepressant-like effects and improved cognitive impairments induced by chronic stress in rats. In another study on a group of depressive middle-aged Korean females showed that they had lower flavonoid intake, including eriodictyol (Park, Jaiswal, and Lee, 2021).

## 9 Mechanisms of anti-depressant action of flavonoids

**FIGURE 8:**
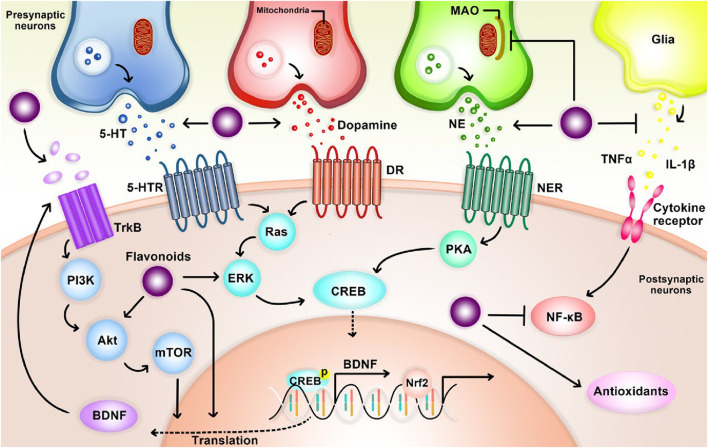
The overall mechanism of flavonoids on depression.

Preclinical studies suggest antidepressant properties in naturally occurring flavonoids, though the mechanisms remain unclear. One proposed mechanism involves functional mimicry of conventional antidepressants. Flavonoids may achieve this by: 1) inhibiting excessive apoptosis (via modulation of caspase-3, -9, Bax, and Bak protein expression), 2) modulating behavioral patterns, cytokine levels, and oxidative stress, and 3) influencing cellular energy metabolism (Hritcu et al., 2017a). Notably, their antioxidant properties are believed to be critical for both antidepressant and neuroprotective effects (Hritcu et al., 2017a; Ko et al., 2020). Flavonoids likely exert their effects through diverse pathways, including modulation of neurotransmission receptors, brain-derived neurotrophic factor (BDNF) levels, and neuronal growth. They may also inhibit enzymes like monoamine oxidase (MAO) and acetylcholinesterase, regulate calcium and potassium ion channels, and promote brain plasticity and mitochondrial health (Hritcu et al., 2017a) (Figure 8).

## 10 Flavonoids and neurotransmitters

Animal studies suggest various flavonoids have anti-inflammatory, antidepressant, and antioxidant properties. These effects seem to be due to the flavonoids influencing neurotransmitter levels in the brain through interactions with cellular components like transcription factors, enzymes, and kinases (Hritcu et al., 2017a). Specific flavonoids, including rutin, quercetin, and luteolin, may act on neurotransmitters directly or through their receptors to exert antidepressant effects (Machado et al., 2008; Samad et al., 2018; Yan et al., 2015; Sur and Lee, 2022). Additionally, some flavonoids like cyanidin and quercetin inhibit an enzyme (MAO) that breaks down neurotransmitters, leading to increased brain neurotransmitter levels (Fang et al., 2020; Singh, Chauhan, and Shri, 2021).

## 11 Flavonoids and BDNF expression

Brain-derived neurotrophic factor (BDNF) is a highly abundant protein in the human brain, with additional presence in both blood plasma and serum (Miranda et al., 2019). This neurotrophic factor plays a critical role in maintaining neuronal morphology by protecting dendrites and axons, facilitating synaptic plasticity, and regulating neuronal survival and intracellular signaling pathways (Bathina and Das, 2015). Notably, BDNF has been implicated in the pathophysiology of various neurological and psychological disorders, including depression (Autry and Monteggia, 2012). Recent clinical studies further support this association, demonstrating reduced plasma and serum BDNF levels in patients diagnosed with Major Depressive Disorder (MDD) (Lee and Kim, 2010).

Recent research suggests that the critical role of BDNF in the nervous system is primarily mediated by its binding to the TrkB receptor. Chronic stress, a well-established precipitating factor for depression, can lead to a decrease in BDNF support. This reduction weakens the anti-apoptotic control of BCL-2, ultimately decreasing the survival of neurogenic cells (Razzoli et al., 2011; Liu et al., 2011; Yamada and Nabeshima, 2003). Consequently, this may have detrimental effects on hippocampal function and potentially contribute to the development of depression. Furthermore, BDNF binding to TrkB activates various intracellular signaling pathways, including tyrosine-kinase activity, TrkB autophosphorylation, phospholipase C-gamma, mitogen-activated protein kinase, and phosphatidyl-inositol 3-kinase pathways (Razzoli et al., 2011; Yamada and Nabeshima, 2003). Furthermore, activation of the CREB response element at Ser133 of the CREB protein leads to upregulation of BCL-2 and BDNF gene expression. This contributes to enhanced neurogenesis, synaptic plasticity, and promotes neuronal survival (Razzoli et al., 2011; Liu et al., 2011; Yamada and Nabeshima, 2003; Jin, 2020; Ginsberg et al., 2019). Additionally, the BDNF-TrkB signaling pathway not only supports neuronal development and survival but also promotes the growth of dendritic spines. These spines provide the structural foundation for synapse formation, ultimately improving synaptic transmission efficiency (Razzoli et al., 2011; Liu et al., 2011; Yamada and Nabeshima, 2003; Jin, 2020; Ginsberg et al., 2019; Miao, Wang, and Sun, 2020).

Preclinical studies have demonstrated the antidepressant potential of various isolated flavonoids through their ability to reverse reductions in BDNF levels and promote its expression (Hritcu et al., 2017a). These flavonoids include phlioridzin, cyaniding, icariin, quercetin, hyperoside, apigenin, baicalein, chrysin, 7,8-dihydroxyflavone, hesperidin, naringenin. In rodent models, these flavonoids have been shown to increase hippocampal BDNF levels, modulate neuronal networks, maintain brain plasticity, and regulate neurogenesis, neuronal maturation, and synaptogenesis (Holzmann et al., 2015; Neshatdoust et al., 2016; Li et al., 2016b; Sharma, Kumar, and Singh, 2019).

## 12 Flavonoids and oxidative stress

Several compounds, particularly flavonoids with their potent antioxidant activity, are categorized as suppressors of oxidative stress, protecting the body from reactive oxygen species (ROS) (Pietta, 2000). While the precise mechanisms and sequence of events by which these free radicals disrupt cellular function remain unclear, lipid peroxidation appears to be a key mechanism leading to cellular membrane damage (Pizzino et al., 2017; Birben et al., 2012). Organisms have evolved various mechanisms to combat cellular damage caused by free radicals. These antioxidant defense mechanisms include enzymatic components such as catalase, glutathione peroxidase, and superoxide dismutase, alongside non-enzymatic counterparts like tocopherol, glutathione, and ascorbic acid (Kurutas, 2015; Lee KyungHee et al., 2020; Lobo et al., 2010). Notably, flavonoids have been shown to exhibit synergistic and additive effects with these endogenous scavengers. With their potent antioxidant activity, flavonoids can interfere with multiple free radical-generating systems simultaneously, thereby enhancing the action of endogenous antioxidants. This ultimately results in reduced cellular disruption and apoptosis (Hritcu et al., 2017a).

In the pathophysiology of depression, low antioxidant levels and high free radical concentrations contribute to lipid peroxidation, DNA strand breaks, enzyme inactivation, and ultimately, neuronal damage (Pizzino et al., 2017; Birben et al., 2012). Notably, some flavonoids can directly scavenge superoxide radicals, while others target highly reactive peroxynitrite species, representing a major mechanism of their action (Panche, Diwan, and Chandra, 2016c; Pietta, 2000; Kumar, Gupta, and Pandey, 2013). Additionally, flavonoids may exert their protective effects through interactions with various enzymes. These multifaceted properties position flavonoids as promising bioactive molecules for preventing oxidative stress-induced disorders like depression (Qu et al., 2022; Kumar, Gupta, and Pandey, 2013).

Nitric oxide (NO) is generated by various cell types, including macrophages and endothelial cells (Radi, 2018). In macrophages, elevated levels of nitric oxide synthase lead to increased production of both superoxide ions and NO. The reaction between NO and free radicals forms peroxynitrite, a highly detrimental species that causes oxidative damage within cells (Radi, 2018; Bloodsworth, O’Donnell, and Freeman, 2000). Flavonoids, through their antioxidant properties, can scavenge these free radicals, effectively preventing their interaction with NO and minimizing cellular damage. Interestingly, some studies suggest that NO itself might exhibit radical behavior and can be directly scavenged by certain flavonoids (Hritcu et al., 2017a).

Antioxidant enzymes like catalase, glutathione peroxidase, and superoxide dismutase (SOD) play a crucial role in cellular defense by converting free radicals into less harmful molecules. Among these enzymes, SOD holds particular importance. It catalyzes the conversion of superoxide, a reactive oxygen species, into the comparatively less toxic hydrogen peroxide (H2O2) (Tiedge et al., 1998). Additionally, SOD interacts with other neuroprotective components, further enhancing its protective effects (Ighodaro and Akinloye, 2018). Notably, hesperidin, a specific flavonoid, has been shown to increase the activity of SOD, thereby reducing oxidative stress levels in the brain (Hritcu et al., 2017a).

## 13 Toxicological effects of flavonoids

The toxicological effects of flavonoids have been demonstrated in the literature in a wide range of organ systems. A vast array of complex problems including physiological, physicochemical, and molecular processes should be considered for optimal understanding of the clinical toxicities of flavonoid polyphenols. In a sub-chronic toxicity study conducted by Yao et al. (2021) daily oral administration of chrysin (1,000 mg/kg) showed a major decreased body weight whereas liver weight was increased significantly in male rats. A noticeable alteration in the hematology (Red Blood Cell (RBC), mean corpuscular hemoglobin (MCH), mean corpuscular hemoglobin concentration (MCHC), TLC, lymphocytes, and neutrophil) and blood chemistry (albumin, bilirubin, ALT, AST, creatinine, and Gamma-glutamyltransferase) were observed in chrysin (1,000 mg/kg) treated rats that were either limited to one sex or lacked dose-response or were within the normal laboratory ranges. There was a major increase in hepatic and renal oxido-nitrosative stress in chrysin (1,000 mg/kg) treated rats. There was no significant change in electrocardiographic (except heart rate), hemodynamic, left ventricular function, and lung function test. Renal and hepatic histological aberrations were induced in chrysin (1,000 mg/kg) treated rats.

Besides, several herbal productions rich in flavonoids have been shown to affect the rate of absorption of various drugs when co-administered (Galati and O'Brien, 2004). Studies have identified CYP450 enzymes as a key mechanism through which dietary flavonoids modify the bioavailability of therapeutic drugs. Flavonoid compounds, whether consumed in isolated form or as part of herbal preparations or dietary supplements, were found to inhibit or induce various isoforms of CYP450 enzymes in the gastrointestinal tract and liver, and also to modify xenobiotic efflux in the gut. While this can be beneficial for drugs with low bioavailability or metabolic stability, this pharmacokinetic alteration can have a negative impact on drugs with an extremely narrow therapeutic index, such as captopril, lisinopril, and digoxin (Fasinu, Bouic, and Rosenkranz, 2012). Although flavonoids have been found to induce toxicity, the underlying mechanisms of this toxicity remain largely unknown. Nutraceutical regulations are constantly evolving to address health, pharmaceutical, and safety concerns.

## 14 The strengths and the weakness

The present study includes both *in vivo* and *in vitro* experimental and clinical trials, which can provide a comprehensive overview of the mechanism and function of the most essential and promising flavonoids, resulting in a potential perspective for future researchers. Also, assessing their effects on disease lets us discover the real impact of flavonoids on humans. But the major limitation of the use of flavonoids is the adverse effects associated with their consumption in high doses, including necroptosis, apoptosis, cardiac dysfunction, and neurotoxic side effect (Satari et al., 2021). Also, some flavonoids with higher doses show the ability to direct interaction with deoxyribonucleic acid (DNA) and enhance carcinogen activation into DNA-modifying enzymes (Hodek et al., 2006). Moreover, flavonoids (flavonol) demonstrate antitumor activity in renal carcinoma cells without toxicity effects on normal cells (Vargas et al., 2018). Thus, their potentially harmful effect on the human body should be investigated in detail.

No statistical measures have been taken, and meta-analysis needs to be done in future studies to achieve flavonoids’ therapeutic and toxic dosage. Due to the inherent format of the narrative reviews, quality assessment of the studies has not been done thoroughly; thus, it may demonstrate some biased results. High doses of flavonoids can cause nausea, headaches, tingling in the limbs, or liver toxicity in some people because it is recommended not to take it during pregnancy, and breastfeeding is not established safety in these conditions. It is best to avoid flavonoid supplements. At high doses, flavonoids can act as mutagens, pro-oxidants that generate free radicals, and inhibitors of key enzymes involved in hormone metabolism. A typical vegetarian diet may outweigh the effects and should be used with caution if taken over. Because flavonoids readily cross the placenta, the fetus can be particularly at risk (Skibola and Martyn, 2000).

Also mentioned are these cases, but the number of findings is minimal and more studies need to investigate.

## 15 Conclusion and future directions

As outlined in this paper and Supplementary Table S1, there is evidence supporting the hypothesis that certain natural compounds, such as flavonoids, have antidepressant properties. These compounds can be used as alternative and complementary therapies for depression. A variety of mechanisms are proposed to explain the anti-depressant potential of flavonoids, including modulation of the neurotransmitter system and regulation of dopaminergic, noradrenergic, and serotonergic pathways in the CNS or by neurotrophic factors, anti-inflammatory, etc (as summarized in Table 1). Considering controversies in the results of different studies and side effects of these compounds, more detailed *in vivo* and *in vitro* studies should be conducted to profoundly investigate the potential of flavonoids in the prophylaxis and treatment of psychiatric disorders.

**TABLE 1:** A summary of possible cellular and molecular mechanisms of anti-depressant action of flavonoids.

Chalcones	DHIPC	1. Increased the concentrations of the main neurotransmitters serotonin and noradrenaline in the brain (Zhao et al., 2018) 2. Increased 5-hydroxyindoleacetic acid contents in the hippocampus (Zhao et al., 2018)
	Phlioridzin	1. Enhancing the expression of GSH, BDNF, TrkB, CREB, and ERK (Kamdi, Raval, and Nakhate, 2021)
Anthocyanins	Cyanidin	1. Inhibition of monoamine oxidases activity (Fang et al., 2020) 2. Increased production of BDNF (Fang et al., 2020; Qu et al., 2022) 3. Upregulation of the PI3K/AKT/FoxG1/FGF-2 pathway (Shan et al., 2020) 4. Decrease the production of pro-inflammatory cytokines (Qu et al., 2022) 5. Upregulation of GFAP, GLAST, EAAT2 (Qu et al., 2022)
	Delphinidin	1. Inhibiting oxidative stress (Di Lorenzo et al., 2019)
	Malvidin	1. Maintaining synaptic plasticity by increasing Rac1 expression (Wang et al., 2018)
Flavonols	Rutin	1. Increasing the access of noradrenaline and serotonin in the synaptic cleft (46) 2. Protecting from oxidative stress (Scheggi et al., 2016)
	Kaempferol	1. Enhancement of anti-inflammation effects and antioxidant abilities via upregulation of AKT/β-catenin cascade activity (Gao et al., 2019) 2. Increase of NE, DA, and 5-HT (Yan et al., 2015) 3. Increase of POMC mRNA or plasma β-endorphin level (Park et al., 2010)
	Kaempferol-3-O-_-Dglucose	1. Increase of NE, DA and 5-HT (Yan et al., 2015)
	Kaempferitrin	1. Regulating serotonergic system via interaction with presynaptic 5-HT1A receptors (Cassani et al., 2014) 2. Regulating HPA axis (Cassani et al., 2014)
	Myricetin	1. Improving the activities of glutathione peroxidase (GSH-PX) in the hippocampus (Ma et al., 2015) 2. Reducing plasma corticosterone levels (Ma et al., 2015) 3. Normalize BDNF levels in the hippocampus (Ma et al., 2015)
	Myricitrin	1. Inhibition of nitric oxide (Meyer et al., 2017) 2. Promoting hippocampal neurogenesis (Meyer et al., 2017)
	Morin	1. Probability of influencing the role of the L-arginine-nitric oxide pathway (Ben-Azu et al., 2019) 2. Elevating the epinephrine, norepinephrine, and serotonin levels in both the hippocampus and the cortex (Hassan et al., 2020) 3. Decreasing the tissue levels of inflammatory markers; TNF-alpha, TLR-4, NLRP3, IL-1beta, caspase-1 and caspase-3 levels (Hassan et al., 2020)
	Quercetin	1. Modulation of inflammation (Şahin et al., 2019) 2. Regulating the serotonergic enzymes (Singh, Chauhan, and Shri, 2021; Samad et al., 2018) 3. Preventing brain oxidative stress by inhibiting MAO-A activity (Şahin et al., 2019; Samad et al., 2018; Singh, Chauhan, and Shri, 2021; Scheggi et al., 2016) 4. Producing cholinergic neurotransmissions (Samad et al., 2018) 5. Increasing the expression of BDNF (Hou et al., 2010) 6. Suppressing oxidative/nitrosative stress-mediated neuroinflammation/apoptotic cascade (Rinwa and Kumar 2013) 7. Increase of NE, DA, and 5-HT (Yan et al., 2015) 8. Neuroprotective effects via microglial inhibitory pathway (Rinwa and Kumar 2013)
	Quercetin3-O-D-glucoside	1. Increase of NE, DA and 5-HT (4)
	Icariin	1. Anti-oxidant action (Liu et al., 2015) 2. Inhibiting NF-kB signaling (Liu et al., 2015) 3. Inhibiting NLRP3 -inflammasome/caspase-1/IL-1b axis (Liu et al., 2015) 4. Increasing BDNF expression (Wu et al., 2013; Di et al., 2023; Gong et al., 2016) 5. Inhibiting the production of inflammatory cytokines 6. (TNF-alpha, IL-6, IL-1β) (Wu et al., 2013; Liu et al., 2015; Di et al., 2023) 7. Restoring the glucocorticoid sensitivity (Wu et al., 2013) 8. Regulation of the HPA- axis (Wei et al., 2016) 9. Lowering the expression levels of FKBP5 and SGK1 (Wei et al., 2016) 10. creasing the expression of monoamine neurotransmitters such as 5-HT, dopamine, and norepinephrine (Di et al., 2023) 11. upregulating the relative expression levels of p-TrkB/TrkB, p-Akt/Akt, p-CREB/CREB, MAPK3, MAPK1, Bcl-2, EGFR, and mTOR (Di et al., 2023)
	Hyperoside	1. Activation of D2-DA receptors of the dopaminergic pathway (Haas et al., 2011) 2. inhibiting the NLRP1 inflammasome via CXCL1/CXCR2/BDNF signaling pathway (Song et al., 2022) 3. including increased expression of BDNF and CREB (Zheng et al., 2012) 4. up-regulating the AC–cAMP–CREB signaling pathway (Zheng et al., 2012) 5. Decreasing plasma ACTH and corticosterone concentration (regulation of HPA axis) (Butterweck, Hegger, and Winterhoff, 2004)
	Fisetin	1. Regulation of central serotonin and noradrenaline levels (Zhen et al., 2012) 2. Reversing the overexpression of proinflammatory cytokine (especially, IL-6, IL-1, and TNF-α) (Yu et al., 2016) 3. NF-kB modulation (Yu et al., 2016) 4. Activating the TrkB signaling pathway (Wang et al., 2017) 5. Downregulation of TNF-α/NLRP3 expression (Gopnar et al., 2023)
Isoflavonoids Flavanones	Genistein	1. Down-regulating miR-221/222 (a microRNA that increases in the prefrontal cortex of depressed patients) by targeting connexin 43 (Shen et al., 2018) 2. Regulating the serotonergic enzymes (Hu et al., 2017; Kageyama et al., 2010)
	Daidzein	1. Decrease of stress-related hormones (Chen et al., 2021) 2. Mitigating HPA axis hyperactivity (Chen et al., 2021) 3. Decrease of inflammatory cytokines (Chen et al., 2021)
	Apigenin	1. Inhibition of inflammatory cytokines, iNOS, and COX-2 in the brain (Li et al., 2015; Li et al., 2016b) 2. Regulating dopaminergic mechanisms and their effect on DOPAC, DA, and HVA concentrations in the mice brain (Nakazawa et al., 2003) 3. Increased serum corticosterone and reduction in the level of hippocampal BDNF (Weng et al., 2016) 4. Regulating the serotonergic enzymes (Yi et al., 2008) 5. Increasing autophagy through the AMPK/mTOR pathway (Zhang et al., 2019)
	Baicalein	1. Reversing the reduction of extracellular ERK phosphorylation (Xiong et al., 2011) 2. Enhancing level of hippocampal BDNF expression (Xiong et al., 2011) 3. Promoting hippocampus neurogenesis via upregulating cAMP/PKA pathway (Zhang et al., 2018) 4. Ameliorating neuroinflammation (Du et al., 2019)
	Chrysin	5. Enhancing level of hippocampal BDNF expression (Jesse et al., 2015; Borges Filho et al., 2016a) 6. Increasing Na+,K + -ATPase activity (Jesse et al., 2015) 7. Decreasing ACTH and corticosterone levels (Borges Filho et al., 2016b; Jesse et al., 2015) 8. Inhibiting the production of inflammatory cytokines 9. (TNF-alpha, IL-6, IL-1β) (Borges Filho et al., 2016a; Borges Filho et al., 2016b) 10. Lowering 5-HT1A and 5-HT2A receptors in raphe nucleus and increasing hippocampal 5-HT1A and 5-HT2A (German-Ponciano et al., 2021) 11. interaction with GABAA receptors (Cueto-Escobedo et al., 2020)
	Luteolin	12. Lowering plasma corticosterone and adrenocorticotropic hormone levels (Sur and Lee, 2022) 13. Inhibiting the MAO enzyme, decreasing norepinephrine, and increasing serotonin levels in prefrontal cortex and hippocampus (Sur and Lee, 2022; Cheng et al., 2022; de la Peña et al., 2014) 14. Enhancing synapsin levels (Cheng et al., 2022) 15. Inhibiting inflammation in the hippocampus (Cheng et al., 2022) 16. Suppression of hippocampal expression of stress-related protein of endoplasmic reticulum (Ishisaka et al., 2011) 17. Potentiation of GABA-A receptor-calcium ion channels (de la Peña et al., 2014) 18
	Nobiletin	19. Reversing neuroinflammation (Wang et al., 2020) 20. Promoting autophagy (Wang et al., 2020) 21. Suppressing the activation of NLRP3 inflammasome possibly via the AMPK pathway (Wang et al., 2020) 22. Interaction with noradrenergic, serotonergic, and dopaminergic systems (Yi et al., 2011)
	7,8-Dihydroxyflavone	23. Elevating the expression of BDNF (Zhang et al., 2014) 24. Modulation of nitric oxide signaling pathway (Zhang et al., 2014) 25. Acting as TrkB receptor-specific agonist (Zhang et al., 2016)
	Amentoflavone	1. Interactions with 5-HT2 receptors, α1- and α2-adrenoceptors
Flavanones	Hesperidin	1. Reducing inflammatory cytokine levels (Fu et al., 2019; Antunes et al., 2016; Xie et al., 2020) 2. Suppressing microglia activation (Xie et al., 2020) 3. Inhibiting the l-arginine-NO-cGMP pathway (Donato et al., 2014) 4. Increasing hippocampal BDNF levels (Donato et al., 2014; Antunes et al., 2016; Li et al., 2016a) 5. Inhibition of K+ channels, leading to the inhibition of L-NAME pathway (Donato et al., 2015) 6. Interaction with the k-opioid receptors (Filho et al., 2013) 7. Involvement of serotonergic 5-HT1A receptors (Souza et al., 2013) 8. Activation of the Nrf2/ARE pathway (Zhu et al., 2020) 9. Maintaining brain plasticity (Antunes et al., 2016) 10. Inhibition of acetylcholinesterase activity (Antunes et al., 2016) 11. Increasing ERK phosphorylation (Li, Chen, et al., 2016)
	Naringenin	1. Restoring changes in the kynurenine pathway, the product of tryptophan catabolism as a result of oxido-inflammatory stress (Bansal et al., 2018) 2. Upregulating of SHH, GLI1, NKX2.2, and Paired box protein Pax-6 (PAX6) (Tayyab et al., 2019) 3. Increasing the expression of BDNF in the hippocampus (Tayyab et al., 2019; Yi et al., 2014; Bansal et al., 2018; Zhang et al., 2023; Olugbemide et al., 2021) 4. Inhibiting the production of inflammatory cytokines 5. (TNF-alpha, IL-6, IL-1β) (Bansal et al., 2018; Olugbemide et al., 2021) 6. NF-kB modulation (Bansal et al., 2018; Olugbemide et al., 2021) 7. suppressing microglia activation (Zhang et al., 2023) 8. Involvement of serotonergic and noradrenergic systems (Yi et al., 2010; Yi et al., 2012; Zhang et al., 2023) 9. Anti-oxidative activity (She et al., 2021)
	Naringin	1. Reduction of oxidative damage and regulation of mitochondrial enzyme complex activities (Aggarwal, Gaur, and Kumar 2010) 2. Lowering plasma corticosterone (Kwatra et al., 2016) 3. Modulation of 5-HT1A and kappa-opioid receptors (Kwatra et al., 2016) 4. Inhibiting NMDA receptors in the hippocampus (Wang et al., 2023) 5. Activation of PKA/CREB/BDNF pathway (Wang et al., 2023) 6. Neurogenesis by activating CREB signaling (Gao et al., 2022) 7. Increasing the levels of GAD67 and inhibiting AChE activities (Oladapo et al., 2021) 8. Anti-oxidative activity (Oladapo et al., 2021) 9. Inhibiting the production of inflammatory cytokines (TNF-alpha, IL-6, IL-1β) (Kwatra et al., 2016; Oladapo et al., 2021)

While this review highlights the potential of flavonoids as alternative and complementary therapies for depression, significant research gaps remain. Future studies should prioritize mechanistic investigations in humans to translate preclinical findings. Understanding the specific pathways by which flavonoids exert their antidepressant effects is crucial for optimizing their therapeutic potential.

Based on current knowledge of flavonoid function, future research can leverage this understanding to elucidate mechanisms. For instance, flavonoids are known to possess anti-inflammatory and antioxidant properties. Future studies can explore how these properties contribute to their antidepressant effects, potentially by mitigating neuroinflammation and oxidative stress, both of which are implicated in depression. Additionally, the ability of flavonoids to modulate neurotransmitter systems, including the dopaminergic, noradrenergic, and serotonergic pathways, suggests further investigation into their precise interactions with these systems and how they influence mood regulation.

## Glossary

WHO: World Health Organization
CNS: Central Nervous System
CUMS: Chronic Unpredictable Mild Stress
BDNF: Brain-Derived Neurotrophic Factor
RBC: Red Blood Cell
7,8-DHF: 7,8-Dihydroxyflavone
TrkB: Tropomyosin Receptor Kinase B
TrkA: Tropomyosin Receptor Kinase A
MAO: Monoamine Oxidase
TST: Tail Suspension Test
FST: Porsolt forced Swimming Test
OSFST: Open Space Forced Swim Test
KFL: Kaempferol
AKT: Protein Kinase B
PI3-K: Phosphoinositide 3-Kinase
CSDS: Chronic Social Defeat Stress
GSH-PX: Glutathione Peroxidase
L-NAME: L-Arginine-Nitric Oxide
NOD-like receptor: Nucleotide-binding and Oligomerization Domain, leucine-rich repeat
QE or Quer: Quercetin
RS: Resveratrol
VD: Vas Deferens
UCMS: Unpredictable Chronic Mild Stress
ATP: Adenosine Triphosphate
ACE: Allium Cepa outer scale Extract
EF or EAF: Ethyl acetate Fraction
QG: Quercetin 4′-O-glucoside
TLC: Thin layer chromatography
AMP: Adenosine Monophosphate
cAMP: Cyclic Adenosine Monophosphate
CREB: cAMP-Response Element Binding protein
PSD95: Postsynaptic Density protein 95
Ab: Amyloid-b peptide
AD: Alzheimer’s Disease
PO: Per Oral
IP: Intraperitoneal
miRNA: microRNA
RNA: Ribonucleic Acid
ICR: Institute of Cancer Research
5-HT1A receptors: serotonin 1A receptor
iNOS: inducible Nitric Oxide Synthase
COX-2: Cyclooxygenase-2
LPS: Lipopolysaccharide
NLRP3: NLR family Pyrin domain containing 3
NLR: Neutrophil to Lymphocyte Ratio
PPARγ: Peroxisome proliferator-activated receptor γ
GABA: Gamma-Aminobutyric Acid
sEPSCs: spontaneous postsynaptic excitatory currents
NMDA: N-methyl-d-aspartate
TMCA: 2,4,5-Trimethoxycinnamic Acid
DOPAC: Dihydroxyphenyl Acetic Acid
DA: Dopamine
HVA: Homovanillic Acid
CMS: Chronic Mild Stress
HPA: Hypothalamic-Pituitary-Adrenal
5-HT: Serotonin
5-HIAA: 5-Hydroxy Indole Acetic Acid
LC 3-II/I: Light Chain 3-II/I
AMPK: Adenosine Monophosphate-activated Protein Kinase
ULK1: unc-51 like autophagy activating kinase 1
mTOR: Mammalian Target of Rapamycin
XCHT: Xiao Chai Hu Tang
OFT: Open Field Test
5-HT: 5-hydroxytryptamine
NGF: Nerve Growth Factor
EPK: Extracellular signal-regulated Kinase
TCM: Traditional Chinese Medicine Mechanisms
EA: Echinocystic acid
BLA: basolateral amygdala
PGP-SLN: N-Acetyl Pro–Gly–Pro-solid lipid nanoparticles
OB: Olfactory-Bulbectomized
Glx: Glutamate + Glutamine
TCR: Total Creatine
EAP: experimental autoimmune prostatitis
SLC6A4: Serotonin transporter gene polymorphism
IDO: Indoleamine 2,3 Dioxygenase
BA: Baicalein
NSFT: Novelty Suppressed Feeding Test
HRG: Huangqin (Radix scutellariae), Renshen (Ginseng) and Gancao (Radix glycyrrhizae)
DCX: Doublecortin
RS: Radix Scutellariae
BrdU: 5-Bromo-2-deoxyUridine
NeuN: neuron-specific nuclear protein
PKA: Protein kinase A
DEP: Differentially Expressed Protein
BChE: Butyryl-Cholinesterase
LCPUFA: long-chain omega 3 polyunsaturated fatty acids
BZD: Benzodiazepine
HPLC–SPE–ttNMR: High performance liquid chromatography-solid phase extraction-tube transfer nuclear magnetic resonance
Er: Ergosterol
DMPA: 4-Dimethyl Aminopyridine
EDCI: 3-dimethylamino propyl carbodiimide hydrochloride
ErN: Ergosterol 2-Naphthoate
HMGB1: High-Mobility–Group Box
Chromosomal Protein 1
RAGE: Receptor for Advanced Glycation End-product
NF- κB: Nuclear factor-κB
ARE: Antioxidant Response Element
Nrf2: Nuclear factor erythroid 2-related factor 2
PTSD: Posttraumatic stress disorder
SPS: Single Prolonged Stress
SHH: Sonic Hedgehog
GLI1: Human Glioma-Associated Oncogene Homolog 1
NKX2.2: NK2 Homeobox 2
PAX6: Paired box protein Pax-6
LPS: lipopolysaccharide
POMC: Pro-opiomelanocortin
GSH-PX: Glutathione Peroxidase
IL-1: Interleukin-1
ERK1/2: Extracellular Signal-Regulated Kinases 1 and 2
TBARS: Thiobarbituric acid reactive substances
Glo-1: Glyoxalase 1
qRT-PCR: quantitative real-time PCR
TR-FIA test: time-resolved fluorimetric assay
iTRAQ: isobaric tags for relative and absolute quantification
CRH: corticotropin-releasing hormone
ACTH: adrenocorticotropic hormone
HAM-D: Hamilton Depression Rating Scale
TNF-α: Tumor Necrosis Factor alpha
NF-κB: nuclear factor kappa light chain enhancer of activated B cells
mRNA: messenger ribonucleic acid
HPLC: High-performance Liquid Chromatography
MCH: mean corpuscular hemoglobin
MCHC: Mean Corpuscular Haemoglobin Concentration
DNA: Deoxyribonucleic Acid

## References

[B1] Abdul rahman S. Kadar B. Baharuldin M. Mohd Moklas M. A. 2015. Antidepressant-like effect of methanolic extract of Punica granatum (pomegranate) in mice model of depression, J. Nat. Prod. Biomed. Res., 1, 16–20.

[B2] Aggarwal A. Gaur V. Kumar A. (2010). Nitric oxide mechanism in the protective effect of naringin against post-stroke depression (PSD) in mice. Life Sci. 86, 928–935. 10.1016/j.lfs.2010.04.011 20433854

[B3] Alam F. Mohammadin K. Shafique Z. Amjad S. T. Mohammad Hassham Hassan bin Asad (2022). Citrus flavonoids as potential therapeutic agents: a review. Phytotherapy Res. 36, 1417–1441. 10.1002/ptr.7261 34626134

[B4] Ali S. Corbi G. Maes M. Scapagnini G. Davinelli S. (2021a). Exploring the impact of flavonoids on symptoms of depression: a systematic review and meta-analysis. Antioxidants (Basel) 10, 1644. 10.3390/antiox10111644 34829515 PMC8615051

[B5] Ali S. Corbi G. Maes M. Scapagnini G. Davinelli S. %J. A. (2021b). Exploring the impact of flavonoids on symptoms of depression: a systematic review and meta-analysis. Antioxidants (Basel). 10, 1644. 10.3390/antiox10111644 34829515 PMC8615051

[B6] Al-Ishaq R. K. Abotaleb M. Kubatka P. Kajo K. Dietrich B. (2019). Flavonoids and their anti-diabetic effects: cellular mechanisms and effects to improve blood sugar levels. Biomolecules 9, 430. 10.3390/biom9090430 31480505 PMC6769509

[B7] Amin I. Majid S. Farooq A. Ahmad Wani H. Noor F. Khan R. (2020a). Naringenin (4, 5, 7-trihydroxyflavanone) as a potent neuroprotective agent: from chemistry to medicine. Stud. Nat. Prod. Chem. 65, 271–300. 10.1016/b978-0-12-817905-5.00008-1

[B8] Amin N. Xie S. Tan X. Chen Y. Ren Q. Botchway B. O. A. (2020b). Optimized integration of fluoxetine and 7, 8-dihydroxyflavone as an efficient therapy for reversing depressive-like behavior in mice during the perimenopausal period. Prog. Neuropsychopharmacol. Biol. Psychiatry 101, 109939. 10.1016/j.pnpbp.2020.109939 32243998

[B9] Antunes M. S. Jesse C. R. Ruff J. R. de Oliveira Espinosa D. Gomes N. S. Altvater E. E. T. (2016). Hesperidin reverses cognitive and depressive disturbances induced by olfactory bulbectomy in mice by modulating hippocampal neurotrophins and cytokine levels and acetylcholinesterase activity. Eur. J. Pharmacol. 789, 411–420. 10.1016/j.ejphar.2016.07.042 27460180

[B10] Arteaga-Henríquez G. Simon M. S. Burger B. Weidinger E. Wijkhuijs A. Arolt V. (2019). Low-grade inflammation as a predictor of antidepressant and anti-inflammatory therapy response in MDD patients: a systematic review of the literature in combination with an analysis of experimental data collected in the EU-MOODINFLAME consortium. Front. psychiatry 10, 458. 10.3389/fpsyt.2019.00458 31354538 PMC6630191

[B11] Atteritano M. Mazzaferro S. Bitto A. Cannata M. L. D’Anna R. Squadrito F. (2014). Genistein effects on quality of life and depression symptoms in osteopenic postmenopausal women: a 2-year randomized, double-blind, controlled study. Control. study 25, 1123–1129. 10.1007/s00198-013-2512-5 24114397

[B12] Autry A. E. Monteggia L. M. (2012). Brain-derived neurotrophic factor and neuropsychiatric disorders. Pharmacol. Rev. 64, 238–258. 10.1124/pr.111.005108 22407616 PMC3310485

[B13] Bansal Y. Singh R. Saroj P. Sodhi R. K. Kuhad A. (2018). Naringenin protects against oxido-inflammatory aberrations and altered tryptophan metabolism in olfactory bulbectomized-mice model of depression. Toxicol. Appl. Pharmacol. 355, 257–268. 10.1016/j.taap.2018.07.010 30017640

[B14] Bathina S. Das U. N. (2015). Brain-derived neurotrophic factor and its clinical implications. Archives Med. Sci. 11, 1164–1178. 10.5114/aoms.2015.56342 PMC4697050 26788077

[B15] Ben‐Azu B. Adegbuyi O Aderibigbe Ajayi A. M. Umukoro S. Iwalewa E. O. (2019). Involvement of l‐arginine‐nitric oxide pathway in the antidepressant and memory promoting effects of morin in mice. Drug Dev. Res. 80, 1071–1079. 10.1002/ddr.21588 31407363

[B16] Ben-Azu B. Nwoke E. E. Umukoro S. Aderibigbe A. O. Ajayi A. M. Iwalewa E. O. (2018). Evaluation of the neurobehavioral properties of naringin in Swiss mice. Drug Res. (Stuttg) 68, 465–474. 10.1055/a-0575-3730 29529676

[B17] Birben E. Sahiner U. M. Sackesen C. Erzurum S. Kalayci O. (2012). Oxidative stress and antioxidant defense. World allergy Organ. J. 5, 9–19. 10.1097/WOX.0b013e3182439613 23268465 PMC3488923

[B18] Bloodsworth A. O’Donnell V. B. Freeman B. A. (2000). Nitric oxide regulation of free radical–and enzyme-mediated lipid and lipoprotein oxidation. Arteriosclerosis, thrombosis, Vasc. Biol. 20, 1707–1715. 10.1161/01.atv.20.7.1707 10894807

[B19] Butterweck V. Hegger M. Winterhoff H. (2004). Flavonoids of St. John’s Wort reduce HPA axis function in the rat. Planta medica 70, 1008–1011. 10.1055/s-2004-832631 15490333

[B20] Borges Filho C. Jesse C. R. Donato F. Del Fabbro L. Gomes de Gomes M. Goes A. T. R. (2016a). Chrysin promotes attenuation of depressive-like behavior and hippocampal dysfunction resulting from olfactory bulbectomy in mice. Chemico-Biological Interact. 260, 154–162. 10.1016/j.cbi.2016.11.005 27818124

[B21] Borges Filho C. Jesse C. R. Donato F. Del Fabbro L. Gomes de Gomes M. Goes A. T. R. (2016b). Neurochemical factors associated with the antidepressant-like effect of flavonoid chrysin in chronically stressed mice. Eur. J. Pharmacol. 791, 284–296. 10.1016/j.ejphar.2016.09.005 27609609

[B22] Calvi A. Fischetti I. Verzicco I. Murri M. B. Zanetidou S. Volpi R. (2021). Antidepressant drugs effects on blood pressure. Front. Cardiovasc. Med. 8, 704281. 10.3389/fcvm.2021.704281 34414219 PMC8370473

[B23] Cassani J. Dorantes-Barrón A. M. Novales L. M. Real G. A. Estrada-Reyes R. (2014). Anti-depressant-like effect of kaempferitrin isolated from Justicia spicigera Schltdl (Acanthaceae) in two behavior models in mice: evidence for the involvement of the serotonergic system. Molecules 19, 21442–21461. 10.3390/molecules191221442 25532842 PMC6271707

[B24] Chen L. Wang X. Zhang Y. Zhong H. Wang C. Gao P. (2021). Daidzein alleviates hypothalamic-pituitary-adrenal Axis hyperactivity, ameliorates depression-like behavior, and partly rectifies circulating cytokine imbalance in two rodent models of depression. Front. Behav. Neurosci. 15, 671864. 10.3389/fnbeh.2021.671864 34733143 PMC8559531

[B25] Cheng Y. Wang X. Yu Y. Gu J. Zhao M. Fu Q. (2022). Noise induced depression-like behavior, neuroinflammation and synaptic plasticity impairments: the protective effects of luteolin. Neurochem. Res. 47, 3318–3330. 10.1007/s11064-022-03683-0 35978229

[B26] Choy K. W. Devi Murugan D. Leong X.-F. Abas R. Alias A. Mustafa M. R. (2019). Flavonoids as natural anti-inflammatory agents targeting nuclear factor-kappa B (NFκB) signaling in cardiovascular diseases: a mini review. Front. Pharmacol. 10, 1295. 10.3389/fphar.2019.01295 31749703 PMC6842955

[B27] Cipriani A. Furukawa T. A. Salanti G. Geddes J. R. Higgins J. P. Churchill R. (2009). Comparative efficacy and acceptability of 12 new-generation antidepressants: a multiple-treatments meta-analysis. Lancet 373, 746–758. 10.1016/S0140-6736(09)60046-5 19185342

[B28] Cueto-Escobedo J. Andrade-Soto J. Lima-Maximino M. Maximino C. Hernández-López F. Rodríguez-Landa J. F. (2020). Involvement of GABAergic system in the antidepressant-like effects of chrysin (5, 7-dihydroxyflavone) in ovariectomized rats in the forced swim test: comparison with neurosteroids. Behav. Brain Res. 386, 112590. 10.1016/j.bbr.2020.112590 32184157

[B29] de la Peña J. B. Kim C. A. Lee H. L. Yoon S. Y. Kim H. J. Hong E. Y. (2014). Luteolin mediates the antidepressant-like effects of Cirsium japonicum in mice, possibly through modulation of the GABAA receptor. Arch. Pharm. Res. 37, 263–269. 10.1007/s12272-013-0229-9 23925560

[B30] Dhalaria R. Verma R. Kumar D. Puri S. Tapwal A. Kumar V. (2020). Bioactive compounds of edible fruits with their anti-aging properties: a comprehensive review to prolong human life. Antioxidants 9, 1123. 10.3390/antiox9111123 33202871 PMC7698232

[B31] Di X. Wan M. Bai Y. N. Lu F. Zhao M. Zhang Z. (2023). Exploring the mechanism of Icariin in the treatment of depression through BDNF-TrkB pathway based on network pharmacology. Naunyn Schmiedeb. Arch. Pharmacol. 397, 463–478. 10.1007/s00210-023-02615-1 37470804

[B32] Di Lorenzo A. Sobolev A. P. Nabavi S. F. Sureda A. Moghaddam A. H. Khanjani S. (2019). Antidepressive effects of a chemically characterized maqui berry extract (Aristotelia chilensis (molina) stuntz) in a mouse model of Post-stroke depression. Food Chem. Toxicol. 129, 434–443. 10.1016/j.fct.2019.04.023 31022478

[B33] Donato F. Borges Filho C. Giacomeli R. Alvater E. E. T. Del Fabbro L. Antunes M. da S. (2015). Evidence for the involvement of potassium channel inhibition in the antidepressant-like effects of hesperidin in the tail suspension test in mice. J. Med. food 18, 818–823. 10.1089/jmf.2014.0074 25647144 PMC4492670

[B34] Donato F. Gomes de Gomes M. Goes A. T. R. Borges Filho C. Del Fabbro L. Antunes M. S. (2014). Hesperidin exerts antidepressant-like effects in acute and chronic treatments in mice: possible role of l-arginine-NO-cGMP pathway and BDNF levels. Brain Res. Bull. 104, 19–26. 10.1016/j.brainresbull.2014.03.004 24709058

[B35] Du H.-Xi Chen X.-G. Zhang Li Liu Yi Zhan C.-S. Chen J. (2019). Microglial activation and neurobiological alterations in experimental autoimmune prostatitis-induced depressive-like behavior in mice, Neuropsychiatr. Dis. Treat., 15: 2231–2245. 10.2147/NDT.S211288 31496706 PMC6689565

[B36] Dwyer A. V. Whitten D. L. Hawrelak J. A. (2011). Herbal medicines, other than St. John's Wort, in the treatment of depression: a systematic review. Altern. Med. Rev. 16, 40–49. 21438645

[B37] Fang J.-Li Luo Y. Jin S.-H. Yuan Ke Guo Y. (2020). Ameliorative effect of anthocyanin on depression mice by increasing monoamine neurotransmitter and up-regulating BDNF expression. J. Funct. Foods 66, 103757. 10.1016/j.jff.2019.103757

[B38] Fasinu P. S. Bouic P. J. Rosenkranz B. (2012). An overview of the evidence and mechanisms of herb–drug interactions. Front. Pharmacol. 3, 69. 10.3389/fphar.2012.00069 22557968 PMC3339338

[B39] Filho C. B. Del Fabbro L. de Gomes M. G. Goes A. T. Souza L. C. Boeira S. P. (2013). Kappa-opioid receptors mediate the antidepressant-like activity of hesperidin in the mouse forced swimming test. Eur. J. Pharmacol. 698, 286–291. 10.1016/j.ejphar.2012.11.003 23178563

[B40] Fu H. Liu Li Tong Y. Li Y. Zhang X. Gao X. (2019). The antidepressant effects of hesperidin on chronic unpredictable mild stress-induced mice. Eur. J. Pharmacol. 853, 236–246. 10.1016/j.ejphar.2019.03.035 30928632

[B41] Furukawa T. A. Salanti G. Atkinson L. Z. Leucht S. Ruhe H. G. Turner E. H. (2016). Comparative efficacy and acceptability of first-generation and second-generation antidepressants in the acute treatment of major depression: protocol for a network meta-analysis. BMJ Open 6, e010919. 10.1136/bmjopen-2015-010919 PMC4947714 27401359

[B42] Galati G. O'Brien P. J. (2004). Potential toxicity of flavonoids and other dietary phenolics: significance for their chemopreventive and anticancer properties. Free Radic. Biol. Med. 37, 287–303. 10.1016/j.freeradbiomed.2004.04.034 15223063

[B43] Gao C. Wu M. Du Q. Deng J. Shen J. (2022). Naringin mediates adult hippocampal neurogenesis for antidepression via activating CREB signaling. Front. Cell Dev. Biol. 10, 731831. 10.3389/fcell.2022.731831 35478969 PMC9037031

[B44] Gao W. Wang W. Peng Y. Deng Z. (2019). Antidepressive effects of kaempferol mediated by reduction of oxidative stress, proinflammatory cytokines and up-regulation of AKT/β-catenin cascade. Metab. brain Dis. 34, 485–494. 10.1007/s11011-019-0389-5 30762138

[B45] Gapski A. Gomes T. M. Bredun M. A. Ferreira-Lima N. E. Ludka F. K. Bordignon-Luiz M. T. (2019). Digestion behavior and antidepressant-like effect promoted by acute administration of blueberry extract on mice. Food Res. Int. 125, 108618. 10.1016/j.foodres.2019.108618 31554107

[B46] Garg A. Garg S. Zaneveld L. J. D. Singla A. K. (2001). Chemistry and pharmacology of the citrus bioflavonoid hesperidin. Phytotherapy Res. 15, 655–669. 10.1002/ptr.1074 11746857

[B47] German-Ponciano, Jesús L. Rosas-Sánchez G. U. Ortiz-Guerra S. I. Soria-Fregozo C. Francisco Rodríguez-Landa J. (2021). Effects of chrysin on mRNA expression of 5-ht1a and 5-HT2A receptors in the raphe nuclei and Hippocampus. Rev. Bras. Farmacogn. 31, 353–360. 10.1007/s43450-021-00164-3

[B48] Germán-Ponciano León Jesús Puga-Olguín A. Rovirosa-Hernandez M.De J. Caba M. Meza E. Juan Francisco %J Acta Pharmaceutica Rodríguez-Landa (2020) Differential effects of acute and chronic treatment with the flavonoid chrysin on anxiety-like behavior and Fos immunoreactivity in the lateral septal nucleus in rats, 70, 387–397. 10.2478/acph-2020-0022 32074069

[B49] Ginsberg S. D. Malek-Ahmadi M. H. Alldred M. J. Chen Y. Chen K. Chao M. V. (2019). Brain-derived neurotrophic factor (BDNF) and TrkB hippocampal gene expression are putative predictors of neuritic plaque and neurofibrillary tangle pathology. Neurobiol. Dis. 132, 104540. 10.1016/j.nbd.2019.104540 31349032 PMC6834890

[B50] Golden S. A. Christoffel D. J. Mitra H. Hodes G. E. Magida J. Davis K. (2013). Epigenetic regulation of RAC1 induces synaptic remodeling in stress disorders and depression. Nat. Med. 19, 337–344. 10.1038/nm.3090 23416703 PMC3594624

[B51] Gong M. J. Han B. Wang S. M. Liang S. W. Zou Z. J. (2016). Icariin reverses corticosterone-induced depression-like behavior, decrease in hippocampal brain-derived neurotrophic factor (BDNF) and metabolic network disturbances revealed by NMR-based metabonomics in rats. J. Pharm. Biomed. Anal. 123, 63–73. 10.1016/j.jpba.2016.02.001 26874256

[B52] Gopnar V. V. Rakshit D. Bandakinda M. Kulhari U. Sahu B. D. Mishra A. (2023). Fisetin attenuates arsenic and fluoride subacute co-exposure induced neurotoxicity via regulating TNF-α mediated activation of NLRP3 inflammasome. Neurotoxicology 97, 133–149. 10.1016/j.neuro.2023.06.006 37331635

[B53] Gupta G. Jia Jia T. Yee Woon L. Kumar Chellappan D. Candasamy M. Dua K. (2015). Pharmacological evaluation of antidepressant-like effect of genistein and its combination with amitriptyline: an acute and chronic study. Adv. Pharmacol. Sci. 2015, 164943. 10.1155/2015/164943 26681936 PMC4670631

[B54] Haas J. S. Stolz E. D. Betti A. H. Stein A. C. Schripsema J. Lino von Poser G. (2011). The anti-immobility effect of hyperoside on the forced swimming test in rats is mediated by the D2-like receptors activation. Planta medica 77, 334–339. 10.1055/s-0030-1250386 20945276

[B55] Harmer C. J. Duman R. S. Cowen P. J. (2017). How do antidepressants work? New perspectives for refining future treatment approaches. Lancet Psychiatry 4, 409–418. 10.1016/S2215-0366(17)30015-9 28153641 PMC5410405

[B56] Hassan M.-A. M. Gad A. M. Menze E. T. Badary O. A. El-Naga R. N. (2020). Protective effects of morin against depressive-like behavior prompted by chronic unpredictable mild stress in rats: possible role of inflammasome-related pathways. Biochem. Pharmacol. 180, 114140. 10.1016/j.bcp.2020.114140 32652141

[B57] Higgs J. Wasowski C. Marcos A. Jukič M. Paván C. H. Gobec S. (2019). Chalcone derivatives: synthesis, *in vitro* and *in vivo* evaluation of their anti-anxiety, anti-depression and analgesic effects. Heliyon 5, e01376. 10.1016/j.heliyon.2019.e01376 30949609 PMC6430037

[B58] Hodek P. Hanustiak P. Krízková J. Mikelova R. Krízková S. Stiborová M. (2006). Toxicological aspects of flavonoid interaction with biomacromolecules. Neuro Endocrinol. Lett. 27 (Suppl. 2), 14–17. 17159770

[B59] Holzmann I. Silva L. M. da Silva J. A. C. da Steimbach V. M. B. Marcia Maria de Souza (2015). Antidepressant-like effect of quercetin in bulbectomized mice and involvement of the antioxidant defenses, and the glutamatergic and oxidonitrergic pathways. Pharmacol. Biochem. Behav. 136, 55–63. 10.1016/j.pbb.2015.07.003 26196245

[B60] Hou Y. Aboukhatwa M. A. Lei D.-L. Manaye K. Khan I. Yuan L. (2010). Anti-depressant natural flavonols modulate BDNF and beta amyloid in neurons and hippocampus of double TgAD mice. Neuropharmacology 58, 911–920. 10.1016/j.neuropharm.2009.11.002 19917299 PMC2838959

[B61] Hritcu L. Ionita R. Postu P. A. Gupta G. K. Turkez H. Lima T. C. (2017a). Antidepressant flavonoids and their relationship with oxidative stress. Oxidative Med. Cell. Longev. 2017, 5762172. 10.1155/2017/5762172 PMC5749298 29410733

[B62] Hu P. Ma Li Wang Y.-gui Ye F. Wang C. Zhou W.-H. (2017). Genistein, a dietary soy isoflavone, exerts antidepressant-like effects in mice: involvement of serotonergic system. Neurochem. Int. 108, 426–435. 10.1016/j.neuint.2017.06.002 28606822

[B63] Ighodaro O. M. Akinloye O. A. (2018). First line defence antioxidants-superoxide dismutase (SOD), catalase (CAT) and glutathione peroxidase (GPX): their fundamental role in the entire antioxidant defence grid. Alexandria J. Med. 54, 287–293. 10.1016/j.ajme.2017.09.001

[B64] Ishisaka M. Kakefuda K. Yamauchi M. Tsuruma K. Shimazawa M. Tsuruta A. (2011). Luteolin shows an antidepressant-like effect via suppressing endoplasmic reticulum stress. Biol. Pharm. Bull. 34, 1481–1486. 10.1248/bpb.34.1481 21881237

[B65] Ishola I. O. Chatterjee M. Tota S. Tadigopulla N. Adeyemi O. O. Palit G. (2012). Antidepressant and anxiolytic effects of amentoflavone isolated from Cnestis ferruginea in mice. Pharmacol. Biochem. Behav. 103, 322–331. 10.1016/j.pbb.2012.08.017 22944105

[B66] Islam A. Islam Md S. Rahman Md K. Uddin Md N. Akanda Md R. (2020). The pharmacological and biological roles of eriodictyol. Archives Pharmacal Res. 43, 582–592. 10.1007/s12272-020-01243-0 32594426

[B67] Jesse C. R. Donato F. Giacomeli R. Fabbro L. D. Antunes M. da S. Gomes M. G. De (2015). Chronic unpredictable mild stress decreases BDNF and NGF levels and Na+, K+-ATPase activity in the hippocampus and prefrontal cortex of mice: antidepressant effect of chrysin. Neuroscience 289, 367–380. 10.1016/j.neuroscience.2014.12.048 25592430

[B68] Jia S. Hou Y. Wang D. Zhao X. (2022). Flavonoids for depression and anxiety: a systematic review and meta-analysis. Crit. Rev. Food Sci. Nutr. 63, 8839–8849. 10.1080/10408398.2022.2057914 35400250

[B69] Jin W. (2020). Regulation of BDNF-TrkB signaling and potential therapeutic strategies for Parkinson’s disease. J. Clin. Med. 9, 257. 10.3390/jcm9010257 31963575 PMC7019526

[B70] Jin Y.-S. (2019). Recent advances in natural antifungal flavonoids and their derivatives. Bioorg. and Med. Chem. Lett. 29, 126589. 10.1016/j.bmcl.2019.07.048 31427220

[B71] Kageyama A. Sakakibara H. Zhou W. Yoshioka M. Ohsumi M. Shimoi K. (2010). Genistein regulated serotonergic activity in the hippocampus of ovariectomized rats under forced swimming stress. Biosci. Biotechnol. Biochem. 74, 2005–2010. 10.1271/bbb.100238 20944428

[B72] Kamdi S. P. Raval A. Nakhate K. T. (2021). Phloridzin ameliorates type 2 diabetes-induced depression in mice by mitigating oxidative stress and modulating brain-derived neurotrophic factor. J. Diabetes Metab. Disord. 20, 341–348. 10.1007/s40200-021-00750-1 34178842 PMC8212325

[B73] Khan H. Perviz S. Sureda A. Nabavi S. M. Tejada S. (2018). Current standing of plant derived flavonoids as an antidepressant. Food Chem. Toxicol. 119, 176–188. 10.1016/j.fct.2018.04.052 29704578

[B74] Khan J. Deb P. K. Priya S. Medina K. D. Devi R. Walode S. G. (2021). Dietary flavonoids: cardioprotective potential with antioxidant effects and their pharmacokinetic, toxicological and therapeutic concerns. Molecules 26, 4021. 10.3390/molecules26134021 34209338 PMC8272101

[B75] Khodadadeh A. Hassanpour S. Akbari G. (2020). Effects of hesperidin during pregnancy on antidepressant-like behaviour in postpartum mice. Iran. J. Veterinary Med. 14, 261–270. 10.22059/ijvm.2020.297314.1005062

[B76] Khoo H. E. Azlan A. Tang S. T. Lim S. M. (2017). Anthocyanidins and anthocyanins: colored pigments as food, pharmaceutical ingredients, and the potential health benefits. Food and Nutr. Res. 61, 1361779. 10.1080/16546628.2017.1361779 28970777 PMC5613902

[B77] Ko Y.-H. Kim S.-K. Lee S.-Y. Jang C.-G. (2020). Flavonoids as therapeutic candidates for emotional disorders such as anxiety and depression. Archives Pharmacal Res. 43, 1128–1143. 10.1007/s12272-020-01292-5 33225387

[B78] Kok R. M. Charles F. R. (2017). Management of depression in older adults: a review. Jama 317, 2114–2122. 10.1001/jama.2017.5706 28535241

[B79] Kumar S. Gupta A. Pandey A. K. (2013). Calotropis procera root extract has the capability to combat free radical mediated damage. Int. Sch. Res. Notices 2013, 691372. 10.1155/2013/691372 PMC3809601 24222863

[B80] Kumar S. Pandey A. K. (2013). Chemistry and biological activities of flavonoids: an overview. Sci. world J. 2013, 162750. 10.1155/2013/162750 PMC3891543 24470791

[B81] Kurutas E. B. (2015). The importance of antioxidants which play the role in cellular response against oxidative/nitrosative stress: current state. Nutr. J. 15, 71–22. 10.1186/s12937-016-0186-5 PMC4960740 27456681

[B82] Kwatra M. Jangra A. Mishra M. Sharma Y. Ahmed S. Ghosh P. (2016). Naringin and sertraline ameliorate doxorubicin-induced behavioral deficits through modulation of serotonin level and mitochondrial complexes protection pathway in rat Hippocampus. Neurochem. Res. 41, 2352–2366. 10.1007/s11064-016-1949-2 27209303

[B83] Lee B. Choi G. M. Sur B. (2020a). Antidepressant-like effects of hesperidin in animal model of post-traumatic stress disorder. Chin. J. Integr. Med. 1-8. 10.1007/s11655-020-2724-4 32445019

[B84] Lee B. Sur B. Park J. Kim S.-H. Kwon S. Yeom M. Dae-hyun %J The Korean journal of physiology Hahm, pharmacology: official journal of the Korean Physiological Society, and the Korean Society of Pharmacology. (2013). Chronic administration of baicalein decreases depression-like behavior induced by repeated restraint stress in rats, Korean J. Physiol. Pharmacol. 17: 393–403. 10.4196/kjpp.2013.17.5.393 24227939 PMC3823951

[B85] Lee B.-H. Kim Y.-Ku (2010). The roles of BDNF in the pathophysiology of major depression and in antidepressant treatment. Psychiatry investig. 7, 231–235. 10.4306/pi.2010.7.4.231 PMC3022308 21253405

[B86] Lee K. H. Cha M. Lee B. H. (2020b). Neuroprotective effect of antioxidants in the brain. Int. J. Mol. Sci. 21, 7152. 10.3390/ijms21197152 32998277 PMC7582347

[B87] Li C. F. Chen S. M. Chen X. M. Mu R. H. Wang S. S. Geng D. (2016a). ERK-dependent brain-derived neurotrophic factor regulation by hesperidin in mice exposed to chronic mild stress. Brain Res. Bull. 124, 40–47. 10.1016/j.brainresbull.2016.03.016 27018164

[B88] Li R. Wang X. Qin T. Qu R. Shiping %J Behavioural brain research Ma. (2016b). Apigenin ameliorates chronic mild stress-induced depressive behavior by inhibiting interleukin-1β production and NLRP3 inflammasome activation in the rat brain, Behav. Brain Res., 296: 318–325. 10.1016/j.bbr.2015.09.031 26416673

[B89] Li R. Zhao Di Qu R. Fu Q. Shiping %J Neuroscience letters Ma. (2015). The effects of apigenin on lipopolysaccharide-induced depressive-like behavior in mice, Neurosci. Lett., 594: 17–22. 10.1016/j.neulet.2015.03.040 25800110

[B90] Liu B. Xu C. Wu X. Liu F. Du Y. Sun J. (2015). Icariin exerts an antidepressant effect in an unpredictable chronic mild stress model of depression in rats and is associated with the regulation of hippocampal neuroinflammation. Neuroscience 294, 193–205. 10.1016/j.neuroscience.2015.02.053 25791226

[B91] Liu W. Kinnefors A. Boström M. Rask-Andersen H. (2011). Expression of TrkB and BDNF in human cochlea—an immunohistochemical study. Cell tissue Res. 345, 213–221. 10.1007/s00441-011-1209-3 21739244

[B92] Lobo V. Patil A. Phatak A. Chandra N. (2010). Free radicals, antioxidants and functional foods: impact on human health. Pharmacogn. Rev. 4, 118–126. 10.4103/0973-7847.70902 22228951 PMC3249911

[B93] Ma Z. Wang G. Cui L. Wang Q. (2015). Myricetin attenuates depressant-like behavior in mice subjected to repeated restraint stress. Int. J. Mol. Sci. 16, 28377–28385. 10.3390/ijms161226102 26633366 PMC4691049

[B94] Machado D. G. Luis E. B. B. Cunha M. P. Rs Santos A. Pizzolatti M. G. Brighente I. M. C. (2008). Antidepressant-like effect of rutin isolated from the ethanolic extract from Schinus molle L. in mice: evidence for the involvement of the serotonergic and noradrenergic systems. Eur. J. Pharmacol. 587, 163–168. 10.1016/j.ejphar.2008.03.021 18457827

[B95] Meyer E. Mori M. A. Campos A. C. Andreatini R. Guimarães F. S. Milani H. (2017). Myricitrin induces antidepressant-like effects and facilitates adult neurogenesis in mice. Behav. Brain Res. 316, 59–65. 10.1016/j.bbr.2016.08.048 27569185

[B96] Miao Z. Wang Y. Sun Z. (2020). The relationships between stress, mental disorders, and epigenetic regulation of BDNF. Int. J. Mol. Sci. 21, 1375. 10.3390/ijms21041375 32085670 PMC7073021

[B97] Miranda M. Facundo Morici J. Zanoni M. B. Pedro B. (2019). Brain-derived neurotrophic factor: a key molecule for memory in the healthy and the pathological brain. Front. Cell. Neurosci. 13, 363. 10.3389/fncel.2019.00363 31440144 PMC6692714

[B98] Moon Y. J. Wang X. Morris M. E. (2006). Dietary flavonoids: effects on xenobiotic and carcinogen metabolism. Toxicol. vitro 20, 187–210. 10.1016/j.tiv.2005.06.048 16289744

[B99] Nadar J. S. Popatrao Kale P. Kerunath Kadu P. Prabhavalkar K. Dhangar R. (2018). Potentiation of antidepressant effects of agomelatine and bupropion by hesperidin in mice. Neurology Res. Int. 2018, 9828639. 10.1155/2018/9828639 PMC6230398 30510800

[B100] Nakazawa T. Yasuda T. Ueda J. Bulletin P. Keisuke %J Biological Ohsawa. (2003). Antidepressant-like effects of apigenin and 2,4,5-trimethoxycinnamic acid from Perilla frutescens in the forced swimming test, Biol. Pharm. Bull., 26: 474–480. 10.1248/bpb.26.474 12673028

[B101] Neshatdoust S. Saunders C. Castle S. M. Vauzour D. Williams C. Butler L. (2016). High-flavonoid intake induces cognitive improvements linked to changes in serum brain-derived neurotrophic factor: two randomised, controlled trials. Nutr. healthy aging 4, 81–93. 10.3233/NHA-1615 28035345 PMC5166520

[B102] Nöldner M. Schötz K. (2002). Rutin is essential for the antidepressant activity of *Hypericum perforatum* extracts in the forced swimming test. Planta medica 68, 577–580. 10.1055/s-2002-32908 12142988

[B103] Oladapo O. M. Ben-Azu B. Ajayi A. M. Emokpae O. Eneni A. O. Omogbiya I. A. (2021). Naringin confers protection against psychosocial defeat stress-induced neurobehavioral deficits in mice: involvement of glutamic acid decarboxylase isoform-67, oxido-nitrergic stress, and neuroinflammatory mechanisms. J. Mol. Neurosci. 71, 431–445. 10.1007/s12031-020-01664-y 32767187

[B104] Olugbemide A. S. Ben-Azu B. Bakre A. G. Ajayi A. M. Femi-Akinlosotu O. Umukoro S. (2021). Naringenin improves depressive- and anxiety-like behaviors in mice exposed to repeated hypoxic stress through modulation of oxido-inflammatory mediators and NF-kB/BDNF expressions. Brain Res. Bull. 169, 214–227. 10.1016/j.brainresbull.2020.12.003 33370589

[B105] Organization, World Health (2017). Depression and other common mental disorders: global health estimates. Geneva, Switzerland: World Health Organization.

[B106] Panche A. N. Arvind D. D. Sadanandavalli R. J Journal of nutritional science Chandra. (2016c). Flavonoids: an overview, J. Nutr. Sci., 5, e47, 10.1017/jns.2016.41 28620474 PMC5465813

[B107] Panche A. N. Diwan A. D. SR %J Journal of nutritional science Chandra. (2016b). Flavonoids: an overview, J. Nutr. Sci., 5, e47, 10.1017/jns.2016.41 28620474 PMC5465813

[B108] Panche A. N. Diwan A. D. Chandra S. R. (2016a). Flavonoids: an overview. J. Nutr. Sci. 5, e47. 10.1017/jns.2016.41 28620474 PMC5465813

[B109] Pandey K. B. Rizvi S. I. (2009). Current understanding of dietary polyphenols and their role in health and disease. Curr. Nutr. and Food Sci. 5, 249–263. 10.2174/157340109790218058

[B110] Pannu A. Sharma P. C. Thakur V. K. Goyal R. K. (2021a). Emerging role of flavonoids as the treatment of depression. Biomolecules 11, 1825. 10.3390/biom11121825 34944471 PMC8698856

[B111] Pannu A. Sharma P. C. Thakur V. K. Goyal R. K. (2021b). Emerging role of flavonoids as the treatment of depression. Biomolecules 11, 1825. 10.3390/biom11121825 34944471 PMC8698856

[B112] Park S.-H. Sim Y.-B. Han P.-L. Lee J.-K. Suh H.-W. (2010). Antidepressant-like effect of kaempferol and quercitirin, isolated from opuntia ficus-indica var. saboten. Exp. Neurobiol. 19, 30–38. 10.5607/en.2010.19.1.30 22110339 PMC3214795

[B113] Park S.-J. Jaiswal V. Lee H.-J. %J. A. (2021). Dietary intake of flavonoids and carotenoids is associated with anti-depressive symptoms: epidemiological study and *in silico*-Mechanism analysis. Epidemiological Study Silico—Mechanism Analysis 11, 53. 10.3390/antiox11010053 PMC8773076 35052561

[B114] Perez-Caballero L. Torres-Sanchez S. Romero-López-Alberca C. González-Saiz F. Mico J. A. Berrocoso E. (2019). Monoaminergic system and depression. Cell tissue Res. 377, 107–113. 10.1007/s00441-018-2978-8 30627806

[B115] Pietta P.-G. (2000). Flavonoids as antioxidants. J. Nat. Prod. 63, 1035–1042. 10.1021/np9904509 10924197

[B116] Pizzino G. Irrera N. Cucinotta M. Pallio G. Mannino F. Arcoraci V. (2017). Oxidative stress: harms and benefits for human health. Oxidative Med. Cell. Longev. 2017, 8416763. 10.1155/2017/8416763 PMC5551541 28819546

[B117] Preskorn S. H. Drevets W. C. (2009). Neuroscience basis of clinical depression: implications for future antidepressant drug development. J. Psychiatric Practice® 15, 125–132. 10.1097/01.pra.0000348365.96300.07 19339846

[B118] Qu D. Ye Z. Zhang W. Dai B. Chen G. Wang L. (2022). Cyanidin chloride improves LPS-induced depression-like behavior in mice by ameliorating hippocampal inflammation and excitotoxicity. ACS Chem. Neurosci. 13, 3023–3033. 10.1021/acschemneuro.2c00087 36254458

[B119] Radi R. (2018). Oxygen radicals, nitric oxide, and peroxynitrite: redox pathways in molecular medicine. Proc. Natl. Acad. Sci. 115, 5839–5848. 10.1073/pnas.1804932115 29802228 PMC6003358

[B120] Ramesh P. Jagadeesan R. Sekaran S. Dhanasekaran A. Vimalraj S. (2021). Flavonoids: classification, function, and molecular mechanisms involved in bone remodelling. Front. Endocrinol. 12, 779638. 10.3389/fendo.2021.779638 PMC8649804 34887836

[B121] Rani A. Anand A. Kumar K. Kumar V. (2019). Recent developments in biological aspects of chalcones: the odyssey continues. Expert Opin. Drug Discov. 14, 249–288. 10.1080/17460441.2019.1573812 30773996

[B122] Rao P. V. Krishnan K. T. Salleh N. Gan S. H. (2016). Biological and therapeutic effects of honey produced by honey bees and stingless bees: a comparative review. Rev. Bras. Farmacogn. 26, 657–664. 10.1016/j.bjp.2016.01.012

[B123] Razzoli M. Domenici E. Carboni L. Rantamaki T. Lindholm J. Castren E. (2011). A role for BDNF/TrkB signaling in behavioral and physiological consequences of social defeat stress. Genes, Brain Behav. 10, 424–433. 10.1111/j.1601-183X.2011.00681.x 21272243

[B124] Rinwa P. Kumar A. (2013). Quercetin suppress microglial neuroinflammatory response and induce antidepressent-like effect in olfactory bulbectomized rats. Neuroscience 255, 86–98. 10.1016/j.neuroscience.2013.09.044 24095694

[B125] Rodríguez-Landa J. F. German-Ponciano L. J. Puga-Olguín A. Jerónimo O. Molecules Olmos-Vázquez J. (2022). Pharmacological, neurochemical, and behavioral mechanisms underlying the anxiolytic- and antidepressant-like effects of flavonoid chrysin, Molecules, 27: 3551, 10.3390/molecules27113551 35684488 PMC9182416

[B126] Rothmore J. (2020). Antidepressant-induced sexual dysfunction. Med. J. Aust. 212, 329–334. 10.5694/mja2.50522 32172535

[B127] Şahin T. D. Selcen Gocmez S. Duruksu G. Yazir Y. Utkan T. (2019). Resveratrol and quercetin attenuate depressive-like behavior and restore impaired contractility of vas deferens in chronic stress-exposed rats: involvement of oxidative stress and inflammation. Naunyn-Schmiedeberg's Archives Pharmacol. 393, 761–775. 10.1007/s00210-019-01781-5 31836917

[B128] Samad N. Saleem A. Yasmin F. Shehzad M. A. (2018). Quercetin protects against stress-induced anxiety-and depression-like behavior and improves memory in male mice, Physiol. Res., Physiological Res., 67, 795, 808. 10.33549/physiolres.933776 30044120

[B129] Santarsieri D. Schwartz T. L. (2015). Antidepressant efficacy and side-effect burden: a quick guide for clinicians. Drugs context 4, 212290. 10.7573/dic.212290 26576188 PMC4630974

[B130] Satari A. Ghasemi S. Habtemariam S. Asgharian S. Lorigooini Z. (2021). Rutin: a flavonoid as an effective sensitizer for anticancer therapy; insights into multifaceted mechanisms and applicability for combination therapy. Evidence-Based Complementary Altern. Med. 2021, 9913179. 10.1155/2021/9913179 PMC8416379 34484407

[B131] Scheggi S. Marandino A. Del Monte D. De Martino L. Pelliccia T. Del Rosario Fusco M. (2016). The protective effect of Hypericum connatum on stress-induced escape deficit in rat is related to its flavonoid content. Pharm. Biol. 54, 1782–1792. 10.3109/13880209.2015.1127979 26731632

[B132] Setchell K. D. R. Brown N. M. Lydeking-Olsen E. (2002). The clinical importance of the metabolite equol—a clue to the effectiveness of soy and its isoflavones. J. Nutr. 132, 3577–3584. 10.1093/jn/132.12.3577 12468591

[B133] Sforzini L. Worrell C. Kose M. Anderson I. M. Aouizerate B. Arolt V. (2022). A Delphi-method-based consensus guideline for definition of treatment-resistant depression for clinical trials. Mol. Psychiatry 27, 1286–1299. 10.1038/s41380-021-01381-x 34907394 PMC9095475

[B134] Shan X. Chen J. Dai S. Wang J. Huang Z. Lv Z. (2020). Cyanidin-related antidepressant-like efficacy requires PI3K/AKT/FoxG1/FGF-2 pathway modulated enhancement of neuronal differentiation and dendritic maturation. Phytomedicine 76, 153269. 10.1016/j.phymed.2020.153269 32593103

[B135] Sharma P. Kumar A. Singh D. (2019). Dietary flavonoids interaction with CREB-BDNF pathway: an unconventional approach for comprehensive management of epilepsy. Curr. Neuropharmacol. 17, 1158–1175. 10.2174/1570159X17666190809165549 31400269 PMC7057203

[B136] She B. Wu H. Xie Q. Zhang M. Zhou N. Pei D. (2021). The effects of methylated flavonoids on depression-like activity and pro-inflammatory cytokine thresholds in mice induced by repeated finasteride administration. Eur. J. Inflamm. 19, 205873922110476. 10.1177/20587392211047646

[B137] Shen F. Huang W.-li Xing B.-ping Xiang F. Feng M. Jiang C.-ming (2018). Genistein improves the major depression through suppressing the expression of Mir-221/222 by targeting connexin 43. Psychiatry investig. 15, 919–925. 10.30773/pi.2018.06.29 PMC6212704 30205672

[B138] Shukla R. Pandey V. Vadnere G. P. Lodhi S. (2019). “'Role of flavonoids in management of inflammatory disorders,” in Bioactive food as dietary interventions for arthritis and related inflammatory diseases (Elsevier).

[B139] Singh V. Chauhan G. Shri R. (2021). Anti-depressant like effects of quercetin 4'-O-glucoside from Allium cepa via regulation of brain oxidative stress and monoamine levels in mice subjected to unpredictable chronic mild stress. Nutr. Neurosci. 24, 35–44. 10.1080/1028415X.2019.1587247 31368414

[B140] Skibola C. F. Martyn T. (2000). J Free radical biology Smith, and medicine. Potential health impacts excessive flavonoid intake 29, 375–383. 10.1016/s0891-5849(00)00304-x 11035267

[B141] Sommer H. Harrer G. (1994). Placebo-controlled double-blind study examining the effectiveness of an hypericum preparation in 105 mildly depressed patients. J. Geriatr. Psychiatry Neurol. 7 (Suppl. 1), S9–S11. 10.1177/089198879400700104 7857516

[B142] Song A. Wu Z. Zhao W. Shi W. Cheng R. Jiang J. (2022). The role and mechanism of hyperoside against depression-like behavior in mice via the NLRP1 inflammasome. Med. Kaunas. 58, 1749. 10.3390/medicina58121749 PMC9788057 36556951

[B143] Souza L. C. de Gomes M. G. André T. R. G. Fabbro L. D. Carlos Filho B. Boeira S. P. (2013). Evidence for the involvement of the serotonergic 5-HT1A receptors in the antidepressant-like effect caused by hesperidin in mice. Prog. Neuro-Psychopharmacology Biol. Psychiatry 40, 103–109. 10.1016/j.pnpbp.2012.09.003 22996046

[B144] Starchenko G. Hrytsyk A. Raal A. Koshovyi О. (2020). Phytochemical profile and pharmacological activities of water and hydroethanolic dry extracts of calluna vulgaris (L.) hull. Herb. Plants (Basel) 9, 751. 10.3390/plants9060751 32549372 PMC7356365

[B145] Sur B. Lee B. (2022). Luteolin reduces fear, anxiety, and depression in rats with post-traumatic stress disorder. Anim. Cells Syst. Seoul. 26, 174–182. 10.1080/19768354.2022.2104925 36046028 PMC9423864

[B146] Suseem S. R. Dhanish J. (2019). The myth and the fact on naringin-A review. Res. J. Pharm. Technol. 12, 367–374. 10.5958/0974-360x.2019.00067.2

[B147] Taheri Y. Rasul Suleria H. A. Martins N. Sytar O. Beyatli A. Yeskaliyeva B. (2020). Myricetin bioactive effects: moving from preclinical evidence to potential clinical applications. BMC complementary Med. Ther. 20, 241–314. 10.1186/s12906-020-03033-z PMC7395214 32738903

[B148] Tayyab M. Farheen S. Khanam N. Hossain M. M. Shahi M. H. Shahi M. H. (2019). Antidepressant and neuroprotective effects of naringenin via sonic hedgehog-GLI1 cell signaling pathway in a rat model of chronic unpredictable mild stress. Neuromolecular Med. 21, 250–261. 10.1007/s12017-019-08538-6 31037465

[B149] Tiedge M. Lortz S. Munday R. Lenzen S. (1998). Complementary action of antioxidant enzymes in the protection of bioengineered insulin-producing RINm5F cells against the toxicity of reactive oxygen species. Diabetes 47, 1578–1585. 10.2337/diabetes.47.10.1578 9753295

[B150] Vargas F. Romecín P. García-Guillén A. I. Wangesteen R. Vargas-Tendero P. Dolores Paredes M. (2018). Flavonoids in kidney health and disease. Front. Physiology 9, 394. 10.3389/fphys.2018.00394 PMC5928447 29740333

[B151] Wang G. Yang H. Zuo W. Mei X. (2023). Antidepressant-like effect of acute dose of Naringin involves suppression of NR1 and activation of protein kinase A/cyclic adenosine monophosphate response element-binding protein/brain-derived neurotrophic factor signaling in hippocampus. Behav. Pharmacol. 34, 101–111. 10.1097/FBP.0000000000000713 36503881

[B152] Wang H. Guo Y. Qiao Y. Zhang J. Jiang P. (2020). Nobiletin ameliorates NLRP3 inflammasome-mediated inflammation through promoting autophagy via the AMPK pathway. Mol. Neurobiol. 57, 5056–5068. 10.1007/s12035-020-02071-5 32833187

[B153] Wang J. Hodes G. E. Zhang H. Zhang S. Zhao W. Golden S. A. (2018a). Epigenetic modulation of inflammation and synaptic plasticity promotes resilience against stress in mice. Nat. Commun. 9, 477. 10.1038/s41467-017-02794-5 29396460 PMC5797143

[B154] Wang S. M. Han C. Bahk W. M. Lee S. J. Patkar A. A. Masand P. S. (2018b). Addressing the side effects of contemporary antidepressant drugs: a comprehensive review. Chonnam Med. J. 54, 101–112. 10.4068/cmj.2018.54.2.101 29854675 PMC5972123

[B155] Wang Y. Wang B. Lu J. Shi H. Gong S. Wang Y. (2017). Fisetin provides antidepressant effects by activating the tropomyosin receptor kinase B signal pathway in mice. J. Neurochem. 143, 561–568. 10.1111/jnc.14226 28945929

[B156] Wang Yan-Shuo Shen C.-Y. Jiang J.-G. (2019). Antidepressant active ingredients from herbs and nutraceuticals used in TCM: pharmacological mechanisms and prospects for drug discovery. Pharmacol. Res. 150, 104520. 10.1016/j.phrs.2019.104520 31706012

[B157] Wei K. Xu Y. Zhao Z. Wu X. Du Y. Sun J. (2016). Icariin alters the expression of glucocorticoid receptor, FKBP5 and SGK1 in rat brains following exposure to chronic mild stress. Int. J. Mol. Med. 38, 337–344. 10.3892/ijmm.2016.2591 27221032

[B158] Weng L. Guo X. Yang Li Yang X. Yuanyuan %J European journal of pharmacology Han (2016) Apigenin reverses depression-like behavior induced by chronic corticosterone treatment in mice, 774, 50–54. 10.1016/j.ejphar.2016.01.015 26826594

[B159] Woo H. D. Kim J. (2013). Dietary flavonoid intake and risk of stomach and colorectal cancer. World J. gastroenterology WJG 19, 1011–1019. 10.3748/wjg.v19.i7.1011 PMC3581988 23467443

[B160] Wu X. Wu J. Xia S. Li B. Dong J. (2013). Icaritin opposes the development of social aversion after defeat stress via increases of GR mRNA and BDNF mRNA in mice. Behav. Brain Res. 256, 602–608. 10.1016/j.bbr.2013.09.034 24064280

[B161] Wu Z.-Yu Sang L.-X. Bing %J World journal of clinical cases Chang (2020) Isoflavones and inflammatory bowel disease, 8, 2081. 10.12998/wjcc.v8.i11.2081 PMC7281056 32548137

[B162] Xie L. Gu Z. Liu H. Jia B. Wang Y. Cao M. (2020). The anti-depressive effects of hesperidin and the relative mechanisms based on the NLRP3 inflammatory signaling pathway. Front. Pharmacol. 11, 1251. 10.3389/fphar.2020.01251 32922291 PMC7456860

[B163] Xiong Z. Jiang Bo Wu P.-F. Jia T. Shi L.-L. Gu J. Jian-Guo %J Biological Chen (2011). Antidepressant effects of a plant-derived flavonoid baicalein involving extracellular signal-regulated kinases cascade. Biol. Pharm. Bull. 34, 253–259. 10.1248/bpb.34.253 21415537

[B164] Yamada K. Nabeshima T. (2003). Brain-derived neurotrophic factor/TrkB signaling in memory processes. J. Pharmacol. Sci. 91, 267–270. 10.1254/jphs.91.267 12719654

[B165] Yan S.-X. Lang J.-L. Song Y.-Y. Wu Y.-Ze Lv M.-H. Zhao X. (2015). Studies on anti-depressant activity of four flavonoids isolated from Apocynum venetum Linn (Apocynaceae) leaf in mice. Trop. J. Pharm. Res. 14, 2269–2277. 10.4314/tjpr.v14i12.17

[B166] Yang S. Zhu G. (2022). 7, 8-dihydroxyflavone and neuropsychiatric disorders: a translational perspective from the mechanism to drug development. Curr. Neuropharmacol. 20, 1479–1497. 10.2174/1570159X19666210915122820 34525922 PMC9881092

[B167] Yao W. Cheng J. Kandhare A. D. Mukherjee-Kandhare A. A. Bodhankar S. L. Lu G. (2021). Toxicological evaluation of a flavonoid, chrysin: morphological, behavioral, biochemical and histopathological assessments in rats. Drug Chem. Toxicol. 44, 601–612. 10.1080/01480545.2019.1687510 31724432

[B168] Yi L.-T. Li C.-Fu Zhan X. Cui C.-C. Xiao F. Zhou L.-P. (2010). Involvement of monoaminergic system in the antidepressant-like effect of the flavonoid naringenin in mice. Prog. Neuro-Psychopharmacology Biol. Psychiatry 34, 1223–1228. 10.1016/j.pnpbp.2010.06.024 20603175

[B169] Yi L.-T. Li J. Li H.-C. Su D.-X. Quan X.-Bo He X.-C. (2012). Antidepressant-like behavioral, neurochemical and neuroendocrine effects of naringenin in the mouse repeated tail suspension test. Prog. Neuro-Psychopharmacology Biol. Psychiatry 39, 175–181. 10.1016/j.pnpbp.2012.06.009 22709719

[B170] Yi L.-T. Li J.-M. Li Y.-C. Pan Y. Xu Q. Ling-Dong %J Life sciences Kong. (2008). Antidepressant-like behavioral and neurochemical effects of the citrus-associated chemical apigenin, Life Sci. 82: 741–751. 10.1016/j.lfs.2008.01.007 18308340

[B171] Yi L.-T. Liu B.-B. Jing Li Liu L. Liu Q. Geng Di (2014). BDNF signaling is necessary for the antidepressant-like effect of naringenin. Prog. Neuro-Psychopharmacology Biol. Psychiatry 48, 135–141. 10.1016/j.pnpbp.2013.10.002 24121063

[B172] Yi L. T. Xu H. L. Feng J. Zhan X. Zhou L. P. Cui C. C. (2011). Involvement of monoaminergic systems in the antidepressant-like effect of nobiletin. Physiol. Behav. 102, 1–6. 10.1016/j.physbeh.2010.10.008 20951716

[B173] Yousuf B. Gul K. Wani A. A. Singh P. (2016). Health benefits of anthocyanins and their encapsulation for potential use in food systems: a review. Crit. Rev. food Sci. Nutr. 56, 2223–2230. 10.1080/10408398.2013.805316 25745811

[B174] Yu S. Yan H. Zhang Li Shan M. Chen P. Ding A. (2017). A review on the phytochemistry, pharmacology, and pharmacokinetics of amentoflavone, a naturally-occurring biflavonoid. Molecules 22, 299. 10.3390/molecules22020299 28212342 PMC6155574

[B175] Yu X. Jiang X. Zhang X. Chen Z. Xu L. Chen L. (2016). The effects of fisetin on lipopolysaccharide-induced depressive-like behavior in mice. Metab. Brain Dis. 31, 1011–1021. 10.1007/s11011-016-9839-5 27209403

[B176] Yusha’u Y. Muhammad U. Nze M. Egwuma J. Igomu O. Abdulkadir M. (2017). Modulatory role of rutin supplement on open space forced swim test murine model of depression. Niger. J. Physiological Sci. 32, 201–205. 29485642

[B177] Zarghami M. Chabra A. Khalilian A. Ali %J Research Journal of Pharmacognosy Asghar Hoseini (2018). Antidepressant effect of Asperugo procumbens L. Comp. fluoxetine a randomized double blind Clin. trial 5, 15–20.

[B178] Zhang L. Liu C. Mei Y. (2020a). Eriodictyol produces antidepressant-like effects and ameliorates cognitive impairments induced by chronic stress. NeuroReport 31, 1111–1120. 10.1097/WNR.0000000000001525 32881773

[B179] Zhang L. Lu R. R. Xu R. H. Wang H. H. Feng W. S. Zheng X. K. (2023). Naringenin and apigenin ameliorates corticosterone-induced depressive behaviors. Heliyon 9, e15618. 10.1016/j.heliyon.2023.e15618 37215924 PMC10192682

[B180] Zhang L. M. Wang H. L. Zhao N. Chen H. X. Li Y. F. Zhang Y. Z. (2014). Involvement of nitric oxide (NO) signaling pathway in the antidepressant action of the total flavonoids extracted from Xiaobuxin-Tang. Neurosci. Lett. 575, 31–36. 10.1016/j.neulet.2014.04.039 24792392

[B181] Zhang M. W. Zhang S. F. Li Z. H. Han F. (2016). 7,8-Dihydroxyflavone reverses the depressive symptoms in mouse chronic mild stress. Neurosci. Lett. 635, 33–38. 10.1016/j.neulet.2016.10.035 27773794

[B182] Zhang R. Guo L. Ji Z. Li X. Zhang C. Ma Z. (2018). Radix scutellariae attenuates CUMS-induced depressive-like behavior by promoting neurogenesis via cAMP/PKA pathway. Neurochem. Res. 43, 2111–2120. 10.1007/s11064-018-2635-3 30259256

[B183] Zhang X. Bu H. Jiang Y. Sun G. Jiang R. Huang X. Qinan %J Molecular medicine reports Wu. (2019). The antidepressant effects of apigenin are associated with the promotion of autophagy via the mTOR/AMPK/ULK1 pathway, Mol. Med. Rep., 20: 2867–2874. 10.3892/mmr.2019.10491 31322238

[B184] Zhao D. H. Wang Y. C. Zheng L. W. Liu B. Y. Guan L. P. (2018). Antidepressant-like effect of a chalcone compound, DHIPC and its possible mechanism. Iran. J. Pharm. Res. 17, 193–201. 29755551 PMC5937090

[B185] Zhen L. Zhu J. Zhao X. Huang W. An Y. Li S. (2012). The antidepressant-like effect of fisetin involves the serotonergic and noradrenergic system. Behav. Brain Res. 228, 359–366. 10.1016/j.bbr.2011.12.017 22197297

[B186] Zheng M. Liu C. Pan F. Shi D. Zhang Y. (2012). Antidepressant-like effect of hyperoside isolated from Apocynum venetum leaves: possible cellular mechanisms. Phytomedicine 19, 145–149. 10.1016/j.phymed.2011.06.029 21802268

[B187] Zhu X. Liu H. Liu Y. Chen Y. Liu Y. Yin X. (2020). The antidepressant-like effects of hesperidin in streptozotocin‐induced diabetic rats by activating Nrf2/ARE/glyoxalase 1 pathway. Front. Pharmacol. 11, 1325. 10.3389/fphar.2020.01325 32982741 PMC7485173

