# Supplementary material for: Flavonoids against depression: a comprehensive review of literature
Source: Front Pharmacol. 2024 Oct 16;15:1411168. doi: 10.3389/fphar.2024.1411168 (PMC11521854; doi:10.3389/fphar.2024.1411168)
Supplement: Supplementary file 1 [file Table1.docx]

| Type of flavonoid | Model/ Study population | Dose | Duration | Behavioural test | Route of administration | Mode of action | Reference |
| --- | --- | --- | --- | --- | --- | --- | --- |
| Quercetin | 24 albino wistar mice | 20 mg/kg | 14 days | light-dark activity, elevated plus maze, morris water maze and forced swim test | Parenteral | ↑ cholinergic neurotransmissions and impairment ↓ of antioxidant enzymes  immobilization stress significantly decreased  Time spent in open arm significantly increased | (1) |
| Quercetin | 96 albino wistar mice | 30 mg/kg | 5 weeks | locomotor activity test, forced swim test, sucrose consumption test, and sucrose preference test | Parenteral (intra-peritoneal) | ↑ antioxidant and anti-inflammatory activity  NM behavioural change | (2) |
| Quercetin | 24 swiss albino mice | 10 and 20 mg/kg | 7 days | forced swim test, sucrose preference test and open field test | Parenteral | ↓ brain oxidative stress and ↑ serotonin levels (by inhibiting MAO-A function)  a signiﬁcant decrease in immobility time  signiﬁcant increase in a number of line crossing | (3) |
| Kaempferol | 50 C57 mice | 10 and 20 mg/kg | 28 days | sucrose preference test, social interaction test and tail suspension test | Parenteral (intra-peritoneal) | upregulating Akt/β-catenin cascade (↑ antioxidant activity in prefrontal cortex)  Increased social interaction time, and the mobility time | (4) |
| Kaempferol and Quercetin | ICR mice | 30 mg/kg | 14 days | tail suspension test, forced swimming test and rota-rod test | Oral | ↑ POMC mRNA or plasma β-endorphin level  NM behavioural change | (5) |
| Myricetin | 60 C57BL/6 mice | 50 mg/kg | 21 days | forced swim test and tail suspension test | Parenteral (intra-peritoneal) | ↑ GSH-PX activity and normalizing BDNF levels in the hippocampus  Decrease the immobility time | (6) |
| Apigenin | 10 groups (n=10 each group) Male ICR mice | 25-50 mg/kg | 7 days | tail suspension test, open field test | Parenteral (intra-peritoneal) | ↓ production of TNF and pro-inflammatory cytokines including IL-1  increased the sucrose consumption  reduced the immobility duration | (7) |
| Apigenin | 8 groups (n=8/group) Male Sprague-Dawley rats | 20 mg/kg | 3 weeks | Chronic unpredictable mild stress procedure, Sucrose preference test, Open field test | Parenteral ( intra-gastric) | ↑ IL-1 secretion in the prefrontal cortex of CUMS mice  Increased sucrose consumption  Increased locomotor activity | (8) |
| Baicalein | EAP mice | 100 mg/kg | Once per day | open field test, elevated plus  maze, forced swim test, and tail suspension test | Parenteral (subcutaneous) | ↓ depression-like behavior and neuroinflammation  Longer open arm time  Increased time in the center | (9) |
| Baicalein | Male Sprague-Dawley | 10-20-40 mg/kg | 14 days  (twice a day) | forced swim test and changes in brain neurotransmitter levels | Parenteral (intra-peritoneal) | ↓ complex symptoms or treating depression, and depressive symptoms  Decreased immobility time  Restoration in climbing behavior | (10) |
| Genistein | BALB/c mice | 5 mg/kg of body weight per day | 8 weeks | Nest building test and splash test | Parenteral (intra-peritoneal) | ↓ depression and expression of microRNA-221/222 by targeting connexin 43  increase the nest building score  Increase grooming frequency time of grooming  decrease the time of latency | (11) |
| Genistein | 262 postmenopausal  women aged 49 to 67 years | 54 mg per day | 2 years | Italian version of the Short Form-36 (SF-36) questionnaire and Zung Self-rating Depression Scale (ZSDS) | Oral | ↓ depression in postmenopausal women with osteopenia and ↑ health status  Increase in physical activity | (12) |
| Genistein | Male ICR mice (weighting 20-22 g upon arrival) | 5,15 or 45 mg/kg of body weight once per day | 3 weeks | Forced swim test, tail suspension test or locomotor activity test | Oral | ↓ depression and behavioral despair  Reduced immobility time | (13) |
| Genistein | ovariectomized Wistar rats | 0.1-1-10-100 mg/kg of body weight per day | 14 days | Forced swimming test | Oral | ↓ depression (by changing serotonergic metabolism)  Reduced immobility time | (14) |
| Genistein and Daidzein and Glycitein | 202 postmenopausal women aged 60 to 75 years | Soy protein containing 52 mg genistein, 41 mg daidzein, and 6 mg glycitein | 1 year | Assessing plasma genistein levels in the final visit blood sample | Oral | ↑ on two dimensions of the SF 36 (social functioning and role limitations)  NM behavioural change | (15) |
| Genistein | Adult male albino mice (BALB/c strain) weighing 20–30g | 10 mg/kg genistein and 5 , 10 mg/kg amitriptyline | 10 days | Locomotor activity test,tail suspension test ;and forced swim test | Oral | ↑ locomotor activity and ↓ immobility time  increases locomotor activity  Decreased immobility time | (16) |
| Phloridzin | E17 SpragueDawley rats | 30–600 μM | 10-12 days |  | Cellular | ↓ incorporation of 3H-inositol and inhabitation in glycogen synthase kinase-3 | (17) |
| Chalcone-1203 | In groups of six male Balb/e mice | 1, 5, and 10 mg/kg | 30 min | Forced swimming test and the tail suspension test | Intraperitoneal | ↑ concentrations of the essential neurotransmitters, 5-HT and norepinephrine, in the hippocampus, hypothalamus and cortex  Reduced immobility time | (18) |
| Cyanidin | 108 specific pathogen-free female mice | 50,100 or 200 mg/kg | 6 weeks | Forced swimming test ,sucrose preference test (SPT),elevated plus-maze test | Parenteral | ↑ monoamine neurotransmitters (by inhibiting MAO) and BDNF and TrkB and phosphorylation levels of ERK1/2  Increased sucrose preference  Decreased immobility time  Increased open arm time | (19) |
| Several type of flavonoids | Male swiss mice | 200,300 and 400mg/kg | Single dose | Tail suspension test ,open field test | Oral | ↓ oxidative stress and TBARS reactivity  Decreased immobility time | (20) |
| Several type of flavonoids | 20 TgAPP/PS1 mice | 50 mg/kg | 4 months | Tail suspension test,locomotion and Morris water maze | Oral | ↑BDNF level and ↓Amyloid-β oligomer level in the hippocampus  No significant behavioural changes | (21) |
| Naringin | Albino mice (Laca strain) | 50 and 100 mg/kg | 10 days | Inclined beam-walking test,forced-swim test ,assessment of gross behavioral activity (locomotor activity) | Intraperitoneal | ↓nitrite, MDA and oxidized glutathione concentration  reduced immobility time and neurological score  signiﬁcant improvement in locomotor activity | (22) |
| Hesperidin | male Sprague-Dawley  rats | 20, 50, and 100 mg/kg | 14 days | Forced swimming test and open field test | Intraperitoneal | prohibiting MAO-A activity, ↓tryptophan hydroxylase-1 level in the hippocampus  decreased immobility time  significant recovery in the time spent climbing in the FST  significant restoration of grooming behavior | (23) |
| Hesperidin | male Swiss  Albino mice | 0.5, 1 mg/kg | 14 days | Forced swim test ,tail suspension test | Intraperitoneal | ↑ serotonin and dopamine levels in the hippocampus  Decreased immobility | (24) |
| Hesperidin | male adults Swiss mice | 0.1, 0.3 and 1 mg/kg | 21 days | Tail suspension test ,open field test | Administered exactly into the cerebral ventricle | ↑BDNF level and as a result inhibiting the l-arginine-NO-cGMP pathway in the hippocampus  Decreased immobility | (25) |
| Hesperidin | male  adult Swiss mice | 0.03 mg/kg | Single dose | Tail suspension test ,open field test | Intraperitoneal | Inhibiting K+ channels and as a result inhibiting the L-NAME pathway  No significant behavioural change | (26) |
| Hesperidin | male adult Swiss mice | 0.1, 0.3 and 1 mg/kg | Single dose | Forced swimming test and open field test | Intraperitoneal | Interacting with the κ-opioid receptor  No significant behavioural change | (27) |
| Hesperidin | male adult  Swiss mice | 0.1, 0.3 and 1 mg/kg | Single dose | Tail suspension test, forced swimming test, open field test | Oral | interacting with the serotonergic 5-HT1A receptors  Reduced immobility time | (28) |
| Hesperidin | 80 ICR male mice | 100, 200 mg/kg | 44 days | sucrose preference test, tail suspension test, forced swimming test | Oral | ↓inflammatory cytokine levels, ↓the BDNF/TrkB^[[1]](#endnote-1)^ and HMGB1/RAGE /NF-κB signaling pathways.  increased percentage of sucrose solution consumption  reduced immobility  relieved the reduction of locomotor activity  increased number of crossing | (29) |
| Hesperidin | Adult male Sprague Dawley rats | 50 and 150 mg/kg | 10 weeks | open field test, Forced Swimming Test | Oral | activating the Nrf2/ARE pathway, and as a result enhancing Glo-1  Reduced immobility duration  Increased central area stay | (30) |
| Naringenin | BALB/c male mice | 25, 50 and 100 mg/kg | 14 days | forced swim test, open field test, despair test, suspension test, sucrose preference test | Oral | ↑BDNF level, ↓ NF-ҝβ, TNF-α, IL-6 and IL-1β levels, restoring changes in kynurenine pathway  significant decrease in locomotion And rearing  restored decrease sucrose preference | (31) |
| Naringenin | adult male Wistar rats | 50 mg/kg/day | 28 days | Open field test, Force swim test | Oral | Up regulating BDNF, GLI1, Shh, NKX2.2 and PAX2  Reduced immobility  Increased number of crossings | (32) |
| Naringenin | male ICR mice | 10, 20 and 50 mg/kg | Single dose | tail suspension test, forced swimming test | Oral | involving serotonergic and noradrenergic systems  Decreased immobility time | (33) |
| Naringenin | 40 Male ICR mice | 5, 10, 20 mg/kg | 14 days | tail suspension test, forced swimming test | Oral | ↓serum corticosterone level and hippocampal GR mRNA level, ↑DA and 5-HT level  Decreased immobility time | (34) |
| Naringenin | male ICR mice | 5, 10 or 20 mg/kg | 21 days | tail suspension test, Sucrose preference test | Cellular | ↑expression of BDNF in hippocampus through activating BDNF signaling pathway | (35) |
| Naringenin | 84 C57BL/6 male mice | 50 or 100 mg/kg | 14 days | Locomotor activity (open-field test), Sucrose preference test, Forced swimming test, Tail suspension test | Oral | attenuating the increased levels of MDA and increasing GSH, SOD and CAT levels  Decreased immobility time | (36) |
| Hesperidin | The NMRI male(n=12) and virgin female mice(n=40) | 0.1, 0.5 and 1 mg/kg | 12 days | Tail suspension test, Open field test, Forced Swimming Test | Oral | ↓ MDA levels, ↑SOD and GPx levels  Decreased immobility time | (37) |
| Chrysin | female C57B/6J mice | 5 and 20 mg/kg | 28 days | Sucrose preference test, Open-field test, Forced swimming test | Oral | ↑BDNF and NGF in prefrontal cortex and hippocampus, ↑Na+,K+-ATPase activity and ↓corticosterone levels  Prevented reduction in sucrose preference  Decreased immobility period | (38) |
| Chrysin | femaleC57B/6Jmice | 5 and 20 mg/kg | 28 days | Splash test, Rota rod test, Tail suspension test | Oral | Attenuating increased CRH, ACTH and IL-1, IL-6, and TNF-α levels  prevented the decrease in the total time of grooming  prevented the increase in the travelled distance and number of rearing  decrease in the total immobility  prevented the increase in immobility time | (39) |
| Chrysin | male C57B/6J mice | 5 and 20 mg/kg | 14 days | Splash test, Open field test, Forced swimming test | Oral | ↑BDNF, ↑5-HT, attenuating increased lippocampal levels of IL-1β, IL-6 TNF-α, kynurenine and indoleamine-2,3-  dioxygenase activity  prevented the decrease in the total time of grooming  a decrease in the total immobility time  prevented the increase in immobility time | (40) |
| Chrysin | Ovariectomized female Wistar mice | 1 mg/kg | Single dose | Locomotor activity test, Forced swim test | Intraperitoneally | interaction with GABA_A_ receptors  Increased rearing time  Decreased number of crossings  Increased grooming time  Longer latency  Shorter immobility | (41) |
| Chrysin | male Wistar mice | 1,5,10 and 20 mg/kg | 28 days | forced swim tests, locomotor activity test | Oral | ↓ 5-HT1A and 5-HT2A receptors in raphe nucleus and ↑ hippocampal 5-HT1A and 5-HT2A  ↑number of crossings  ↑grooming  increased the latency  significantly ↓the total time of immobility respect | (42) |

**Supplementary Table: Antidepressant Properties of Flavonoids**

**References**

1. Samad N, Saleem A, Yasmin F, Shehzad M. Quercetin protects against stress-induced anxiety-and depression-like behavior and improves memory in male mice. Physiological Research. 2018;67(5):795-808.

2. Şahin TD, Gocmez SS, Duruksu G, Yazir Y, Utkan T. Resveratrol and quercetin attenuate depressive-like behavior and restore impaired contractility of vas deferens in chronic stress-exposed rats: involvement of oxidative stress and inflammation. Naunyn-Schmiedeberg's Archives of Pharmacology. 2019:1-15.

3. Singh V, Chauhan G, Shri R. Anti-depressant like effects of quercetin 4'-O-glucoside from Allium cepa via regulation of brain oxidative stress and monoamine levels in mice subjected to unpredictable chronic mild stress. Nutritional Neuroscience. 2019:1-10.

4. Gao W, Wang W, Peng Y, Deng Z. Antidepressive effects of kaempferol mediated by reduction of oxidative stress, proinflammatory cytokines and up-regulation of AKT/β-catenin cascade. Metabolic brain disease. 2019;34(2):485-94.

5. Park S-H, Sim Y-B, Han P-L, Lee J-K, Suh H-W. Antidepressant-like Effect of Kaempferol and Quercitirin, Isolated from Opuntia ficus-indica var. saboten. Experimental Neurobiology. 2010;19(1):30.

6. Ma Z, Wang G, Cui L, Wang Q. Myricetin attenuates depressant-like behavior in mice subjected to repeated restraint stress. International journal of molecular sciences. 2015;16(12):28377-85.

7. Li R, Zhao D, Qu R, Fu Q, Ma SJNl. The effects of apigenin on lipopolysaccharide-induced depressive-like behavior in mice. 2015;594:17-22.

8. Li R, Wang X, Qin T, Qu R, Ma SJBbr. Apigenin ameliorates chronic mild stress-induced depressive behavior by inhibiting interleukin-1β production and NLRP3 inflammasome activation in the rat brain. 2016;296:318-25.

9. Du H-X, Chen X-G, Zhang L, Liu Y, Zhan C-S, Chen J, et al. Microglial activation and neurobiological alterations in experimental autoimmune prostatitis-induced depressive-like behavior in mice. 2019;15:2231.

10. Lee B, Sur B, Park J, Kim S-H, Kwon S, Yeom M, et al. Chronic administration of baicalein decreases depression-like behavior induced by repeated restraint stress in rats. 2013;17(5):393-403.

11. Shen F, Huang W-l, Xing B-p, Fang X, Feng M, Jiang C-m. Genistein improves the major depression through suppressing the expression of Mir-221/222 by targeting connexin 43. Psychiatry investigation. 2018;15(10):919.

12. Atteritano M, Mazzaferro S, Bitto A, Cannata M, D’Anna R, Squadrito F, et al. Genistein effects on quality of life and depression symptoms in osteopenic postmenopausal women: a 2-year randomized, double-blind, controlled study. Osteoporosis international. 2014;25(3):1123-9.

13. Hu P, Ma L, Wang Y-g, Ye F, Wang C, Zhou W-H, et al. Genistein, a dietary soy isoflavone, exerts antidepressant-like effects in mice: Involvement of serotonergic system. Neurochemistry international. 2017;108:426-35.

14. Kageyama A, Sakakibara H, Zhou W, Yoshioka M, Ohsumi M, Shimoi K, et al. Genistein regulated serotonergic activity in the hippocampus of ovariectomized rats under forced swimming stress. Bioscience, biotechnology, and biochemistry. 2010;74(10):2005-10.

15. Kok L, Kreijkamp-Kaspers S, Grobbee DE, Lampe JW, van der Schouw YT. A randomized, placebo-controlled trial on the effects of soy protein containing isoflavones on quality of life in postmenopausal women. Menopause. 2005;12(1):56-62.

16. Gupta G, Jia Jia T, Yee Woon L, Kumar Chellappan D, Candasamy M, Dua K. Pharmacological Evaluation of Antidepressant-Like Effect of Genistein and Its Combination with Amitriptyline: An Acute and Chronic Study. Adv Pharmacol Sci. 2015;2015:164943.

17. Saiardi A, Mudge AWJTp. Lithium and fluoxetine regulate the rate of phosphoinositide synthesis in neurons: a new view of their mechanisms of action in bipolar disorder. 2018;8(1):1-12.

18. Guan L-P, Tang L-M, Pan C-Y, Zhao S-L, Wang S-HJNr. Evaluation of potential antidepressant-like activity of chalcone-1203 in various murine experimental depressant models. 2014;39(2):313-20.

19. Fang J-L, Luo Y, Jin S-H, Yuan K, Guo Y. Ameliorative effect of anthocyanin on depression mice by increasing monoamine neurotransmitter and up-regulating BDNF expression. Journal of Functional Foods. 2020;66:103757.

20. Gapski A, Gomes TM, Bredun MA, Ferreira-Lima NE, Ludka FK, Bordignon-Luiz MT, et al. Digestion behavior and antidepressant-like effect promoted by acute administration of blueberry extract on mice. Food Research International. 2019;125:108618.

21. Hou Y, Aboukhatwa MA, Lei D-L, Manaye K, Khan I, Luo Y. Anti-depressant natural flavonols modulate BDNF and beta amyloid in neurons and hippocampus of double TgAD mice. Neuropharmacology. 2010;58(6):911-20.

22. Aggarwal A, Gaur V, Kumar A. Nitric oxide mechanism in the protective effect of naringin against post-stroke depression (PSD) in mice. Life Sci. 2010;86(25-26):928-35.

23. Lee B, Choi GM, Sur B. Antidepressant-like effects of hesperidin in animal model of post-traumatic stress disorder. Chinese Journal of Integrative Medicine. 2020:1-8.

24. Nadar JS, Kale PP, Kadu PK, Prabhavalkar K, Dhangar R. Potentiation of antidepressant effects of agomelatine and bupropion by hesperidin in mice. Neurology research international. 2018;2018.

25. Donato F, de Gomes MG, Goes ATR, Borges Filho C, Del Fabbro L, Antunes MS, et al. Hesperidin exerts antidepressant-like effects in acute and chronic treatments in mice: possible role of l-arginine-NO-cGMP pathway and BDNF levels. Brain research bulletin. 2014;104:19-26.

26. Donato F, Filho CB, Giacomeli R, Alvater EET, Fabbro LD, Antunes MdS, et al. Evidence for the involvement of potassium channel inhibition in the antidepressant-like effects of hesperidin in the tail suspension test in mice. Journal of medicinal food. 2015;18(7):818-23.

27. Carlos Filho B, Del Fabbro L, de Gomes MG, Goes AT, Souza LC, Boeira SP, et al. Kappa-opioid receptors mediate the antidepressant-like activity of hesperidin in the mouse forced swimming test. European journal of pharmacology. 2013;698(1-3):286-91.

28. Souza LC, de Gomes MG, Goes AT, Del Fabbro L, Carlos Filho B, Boeira SP, et al. Evidence for the involvement of the serotonergic 5-HT1A receptors in the antidepressant-like effect caused by hesperidin in mice. Progress in Neuro-Psychopharmacology and Biological Psychiatry. 2013;40:103-9.

29. Fu H, Liu L, Tong Y, Li Y, Zhang X, Gao X, et al. The antidepressant effects of hesperidin on chronic unpredictable mild stress-induced mice. European journal of pharmacology. 2019;853:236-46.

30. Zhu X, Liu H, Liu Y, Chen Y, Liu Y, Yin X. The Antidepressant-Like Effects of Hesperidin in Streptozotocin‐Induced Diabetic Rats by Activating Nrf2/ARE/Glyoxalase 1 Pathway. Frontiers in Pharmacology. 2020;11:1325.

31. Bansal Y, Singh R, Saroj P, Sodhi RK, Kuhad A. Naringenin protects against oxido-inflammatory aberrations and altered tryptophan metabolism in olfactory bulbectomized-mice model of depression. Toxicology and applied pharmacology. 2018;355:257-68.

32. Tayyab M, Farheen S, Khanam N, Hossain MM, Shahi MH. Antidepressant and neuroprotective effects of naringenin via sonic hedgehog-GLI1 cell signaling pathway in a rat model of chronic unpredictable mild stress. Neuromolecular medicine. 2019;21(3):250-61.

33. Yi L-T, Li C-F, Zhan X, Cui C-C, Xiao F, Zhou L-P, et al. Involvement of monoaminergic system in the antidepressant-like effect of the flavonoid naringenin in mice. Progress in Neuro-Psychopharmacology and Biological Psychiatry. 2010;34(7):1223-8.

34. Yi L-T, Li J, Li H-C, Su D-X, Quan X-B, He X-C, et al. Antidepressant-like behavioral, neurochemical and neuroendocrine effects of naringenin in the mouse repeated tail suspension test. Progress in Neuro-Psychopharmacology and Biological Psychiatry. 2012;39(1):175-81.

35. Yi L-T, Liu B-B, Li J, Luo L, Liu Q, Geng D, et al. BDNF signaling is necessary for the antidepressant-like effect of naringenin. Progress in Neuro-Psychopharmacology and Biological Psychiatry. 2014;48:135-41.

36. She B, Wu H, Xie Q, Zhang M, Zhou N, Pei D, et al. The effects of methylated flavonoids on depression-like activity and pro-inflammatory cytokine thresholds in mice induced by repeated finasteride administration. European Journal of Inflammation. 2021;19:20587392211047646.

37. Khodadadeh A, Hassanpour S, Akbari G. Effects of Hesperidin During Pregnancy on Antidepressant-like behaviour in Postpartum Mice. Iranian Journal of Veterinary Medicine. 2020;14(3):261-70.

38. Jesse C, Donato F, Giacomeli R, Del Fabbro L, da Silva Antunes M, De Gomes M, et al. Chronic unpredictable mild stress decreases BDNF and NGF levels and Na+, K+-ATPase activity in the hippocampus and prefrontal cortex of mice: Antidepressant effect of chrysin. 2015;289:367-80.

39. Borges Filho C, Jesse CR, Donato F, Del Fabbro L, de Gomes MG, Goes ATR, et al. Neurochemical factors associated with the antidepressant-like effect of flavonoid chrysin in chronically stressed mice. 2016;791:284-96.

40. Borges Filho C, Jesse CR, Donato F, Del Fabbro L, de Gomes MG, Goes ATR, et al. Chrysin promotes attenuation of depressive-like behavior and hippocampal dysfunction resulting from olfactory bulbectomy in mice. 2016;260:154-62.

41. Cueto-Escobedo J, Andrade-Soto J, Lima-Maximino M, Maximino C, Hernández-López F, Rodríguez-Landa JFJBBR. Involvement of GABAergic system in the antidepressant-like effects of chrysin (5, 7-dihydroxyflavone) in ovariectomized rats in the forced swim test: comparison with neurosteroids. 2020;386:112590.

42. German-Ponciano LJ, Rosas-Sánchez GU, Ortiz-Guerra SI, Soria-Fregozo C, Rodríguez-Landa JFJRBdF. Effects of Chrysin on mRNA Expression of 5-HT1A and 5-HT2A Receptors in the Raphe Nuclei and Hippocampus. 2021;31(3):353-60.

1. [↑](#endnote-ref-1)
